# HIV-1 virome profiling using HIV-PULSE to guide therapeutic and curative interventions

**DOI:** 10.1016/j.ebiom.2026.106297

**Published:** 2026-05-21

**Authors:** Sofie De Braekeleer, Laurens Lambrechts, Liesbet Termote, Mareva Delporte, Kavita Mehta, Vasiliki Matzaraki, Evy E. Blomme, Philippe Lemey, Bram Vrancken, Casper Rokx, Janneke Stalenhoef, Marvin A.H. Berrevoets, Andre J. van der Ven, Sarah Gerlo, Sofie Rutsaert, Linos Vandekerckhove

**Affiliations:** a HIV Cure Research Center, Department of Internal Medicine and Pediatrics, Ghent University, Ghent, Belgium; b Department of Internal Medicine and Infectious Diseases, Radboudumc, Nijmegen, Netherlands; c Department of Microbiology, Immunology and Transplantation, Rega Institute, Laboratory of Evolutionary and Computational Virology, KU Leuven, Leuven, Belgium; d Department of Internal Medicine and Department of Medical Microbiology and Infectious Diseases, Erasmus University Medical Center, Rotterdam, Netherlands; e Department of Internal Medicine and Infectious Diseases, OLVG, Amsterdam, Netherlands; f Department of Internal Medicine and Infectious Diseases, Elizabeth-Tweesteden Ziekenhuis, Tilburg, Netherlands; g Department of Biomolecular Medicine, Ghent University, Ghent, Belgium; h University Hospital Ghent, Ghent, Belgium

**Keywords:** HIV, Viral reservoir, Long-read sequencing, Drug resistance, Therapy screening

## Abstract

**Background:**

The persistent HIV-1 reservoir remains a major barrier to curing HIV-1, underscoring the need for a detailed understanding of its composition to inform targeted interventions.

**Methods:**

Here, we apply the HIV Proviral Unique molecular identifier-mediated Long-read Sequencing (HIV-PULSE) assay to characterise the viral reservoir in 90 individuals on suppressive antiretroviral therapy. By generating near full-length proviral sequences, HIV-PULSE enables multiple layers of in silico analyses across the HIV-1 genome.

**Findings:**

These data support the construction of participant-specific HIV virome profiles, integrating assessments of proviral intactness, tropism, genetic diversity, archived drug resistance mutations and susceptibility to selected immune-based therapies (broadly neutralising antibodies and HIV-specific immune-mobilising monoclonal T cell receptor therapy IMC-M113V).

**Interpretation:**

Our findings demonstrate that HIV-PULSE provides a comprehensive platform for individualised reservoir profiling, advancing future treatment perspectives and efforts towards an HIV-1 cure.

**Funding:**

This research is part of the 2000HIV study, supported by ViiV Healthcare.

**Research in context**

**Evidence before this study**

People living with HIV-1 (PLWH) can control the virus effectively with antiretroviral therapy (ART). Despite this, a small fraction of infected cells persist, forming a viral reservoir that prevents cure. Understanding the composition of this reservoir and its clinical relevance is essential for developing new therapeutic strategies and curative approaches. Previous studies exploring the viral reservoir have often relied on labour-intensive and costly methods, such as limiting dilution assays, and have often been conducted on relatively small patient cohorts. Recently, HIV-PULSE was developed that allows sequencing of HIV-1 proviral genomes directly from bulk DNA, bypassing the need for limiting dilution and increasing the scalability of reservoir analyses.

**Added value of this study**

This study identifies multiple HIV-1 proviral genomes for 83 individuals using a scalable near full-length HIV-1 proviral genome sequencing assay (HIV-PULSE), providing a detailed viral reservoir profiling. The proviral genomes were used to determine which proviruses were intact, to detect drug resistance mutations, and to predict sensitivity to immune-therapies. In addition, based on the proviral genomes, potential candidates were identified for clinical trials using broadly neutralising antibodies and HIV-specific immune-mobilising monoclonal T cell receptor therapy IMC-M113V. This approach provides a detailed and comprehensive view of the HIV-1 reservoir.

**Implications of all the available evidence**

This study highlights the added value of HIV-PULSE in clinical research, offering a scalable and practical platform for individualised profiling of the HIV-1 reservoir. By combining multiple layers of information in a single workflow, this method improves our understanding of the viral reservoir and could serve as a tool for future clinical studies aiming to target HIV-1 persistence.

## Introduction

Since its discovery over four decades ago, HIV-1 has evolved from a fatal diagnosis into a manageable chronic condition, largely due to the success of antiretroviral therapy (ART). While successfully suppressing plasma viraemia, ART does not eliminate the virus, as HIV-1 establishes a latent reservoir in long-lived memory CD4 T cells that persists even with prolonged treatment, necessitating lifelong adherence to prevent viral rebound.^1^, ^2^, ^3^, ^4^ The presence of this latent reservoir remains a major barrier to cure, yet it also represents a critical target for curative interventions.^5^ A deeper understanding of the composition and diversity of the HIV-1 reservoir is therefore essential to advance curative efforts.

Although ART regimens can be highly effective when taken consistently, drug resistance mutations (DRM) continue to emerge globally due to selective pressure in treated individuals and through transmission at the time of infection.^6^ DRMs pose a significant challenge to the clinical management of people living with HIV-1 (PLWH), contributing to treatment failure, immunologic decline, virologic progression and increased mortality risk.^7^^,^^8^ The problem is particularly concerning for individuals harbouring resistance to multiple antiretroviral drug classes, with studies reporting resistance up to four drug classes.^9^^,^^10^ Once established, viruses with DRMs can become archived within the latent reservoir, further complicating eradication efforts.^11^ While proviral DNA analysis in peripheral blood mononuclear cells (PBMCs) has been explored for over two decades to detect archived DRMs,^12^, ^13^, ^14^ its clinical relevance remains debated due to limited sensitivity and the limitations of bulk PCR methods. Bulk sequencing approaches cannot resolve whether a given read originates from an intact or defective provirus, nor can they resolve the individual proviral genomes that contribute to the signal. Additionally, DRMs resulting from G-to-A hypermutation, rather than from selective drug pressure, are commonly observed in replication-incompetent viruses that harbours premature stop codons and other mutations.^15^ Nevertheless, HIV-1 reservoir genotyping could offer complementary insights into the overall resistance profile of an individual, with implications for both personalised treatment and curative strategies.

Alternative therapeutic strategies are emerging to achieve durable viral control or eliminate infected cells, such as immune-based approaches.^16^ These new strategies involve the use of broadly neutralising antibodies (bNAbs), typically isolated from PLWH who naturally develop a broad serum neutralisation response, that target the HIV-1 envelope glycoprotein.^17^, ^18^, ^19^ Several bNAbs have been extensively characterised for their antiviral efficacy in vitro, in vivo and in clinical studies.^17^^,^^20^, ^21^, ^22^, ^23^, ^24^, ^25^ Despite encouraging results, bNAb therapies face similar obstacles as ART, notably viral resistance and escape mutations.^26^, ^27^, ^28^ Another promising immunological therapy involves immune monoclonal T cell receptor (TCR) therapies, already used in oncology and now adapted for viral infections like hepatitis B and HIV, referred to as immune-mobilising monoclonal T cell receptors against virus (ImmTAV).^29^, ^30^, ^31^ In vitro data showed that an ImmTAV molecule engineered to target an immunodominant human leucocyte antigen (HLA) A∗02:01-resistricted HIV Gag^77–85^ epitope and its seven common escape variants could eliminate latently HIV-infected cells.^31^ A first-in-human phase 1/2 clinical trial is evaluating a bispecific immunotherapy consisting of an anti-CD3 effector arm with TCR binding to HLA-A∗02:01-Gag^77–85^ complexes, IMC-M113V.^32^

Another approach involves targeting the CCR5 coreceptor. A recent study demonstrated that gene-editing approaches, such as the infusion of CCR5-modified autologous CD4 T cells, can be used safely in PLWH.^33^ However, for this strategy to be effective, it is essential to determine the viral tropism. HIV-1 tropism refers to the viral preference for either CCR5 or CXCR4 co-receptors to enter host cells.^34^ Therefore, understanding tropism is crucial for optimising treatment regimens and tailoring personalised therapeutic strategies.

Most current screening methods for DRMs, sensitivity predictions to immune-based therapies, or tropism prediction rely on either short amplicons (<5 kb) of the region of interest or long amplicons sequenced using overlapping Sanger reactions.^25^^,^^35^, ^36^, ^37^ These approaches are labour-intensive, costly and limited in throughput. Recent advances in long-read sequencing technology offer the potential for more comprehensive and scalable profiling of the HIV-1 proviral landscape. The HIV Proviral Unique molecular identifier-mediated Long-read Sequencing (HIV-PULSE) assay addresses these limitations by enabling high-throughput amplification and sequencing of multiple near full-length (NFL) proviral genomes from bulk input in a single reaction.^38^ Unlike bulk screening methods, HIV-PULSE generates individual proviral genomes, allowing clear distinction between intact and defective sequences. In contrast to limiting dilution approaches, which require costly approaches and sequencing each provirus separately, HIV-PULSE leverages Nanopore sequencing technology to achieve higher throughput and cost-efficiency. Benchmarking against the widely used Full-Length Individual Proviral Sequencing (FLIPS) method has demonstrated that HIV-PULSE offers a scalable and reliable platform for NFL proviral sequencing, with estimates suggesting up to a 10-fold reduction in cost compared to FLIPS.^38^^,^^39^

In this study, we applied HIV-PULSE to perform in-depth reservoir characterisation in a subset of participants from the 2000HIV study, a multicentric cohort of ART-suppressed PLWH from the Netherlands.^40^ The high-throughput generation of proviral genomes demonstrates the scalability of HIV-PULSE for large-cohort applications. Using specific bioinformatics tools, we generated participant-specific reservoir profiles, reporting intactness and diversity levels, DRM profiles and in silico predictions of sensitivity to bNAbs and the clinical-stage IMC-M113V. Our findings highlight the added value of HIV-PULSE as a platform for individualised reservoir profiling by analysing key viral regions relevant to HIV-1 cure strategies.

## Methods

### Study population, sample and data collection

This study investigated PLWH from the 2000HIV study within the Human Functional Genomics Project. Extensive details and the design of the 2000HIV study have been previously published.^40^ This prospective longitudinal cohort study recruited 1895 PLWH across four different centres in the Netherlands between October 2019 and October 2021. The following inclusion criteria were taken into account: aged ≥18 years, ≥6 months on ART and a suppressed HIV-RNA viral load of <200 copies/mL. Clinical data, including medical history and HIV-specific characteristics were collected from clinical records and data available from the Dutch national ATHENA cohort.^41^ Sex was self-reported by participants at the time of enrolment.

Blood from the study participants was collected during the baseline study visit via venipuncture and transported overnight at room temperature to the laboratory at Radboudumc, Nijmegen. Isolation of peripheral blood mononuclear cells (PBMC) was done using density gradient separation (Ficoll-paque) in SepMate™ tubes.^42^ PBMC were then shipped to Ghent University and cryopreserved until further processing. Pre-ART plasma Sanger sequences spanning the *pol* region were available for a subset of participants and served as reference sequences for drug resistance analyses. HLA class I typing data were available for participants in the cohort and were used in downstream analyses of Gag-targeted immune responses.

For the current study, we selected 90 participants from the 2000HIV cohort based on a DNA concentration greater than 90 ng/μL and HIV-1 DNA copies input per replicate exceeding three copies per microlitre. All selected participants were confirmed to have HIV-1 subtype B through *env* subtyping, reflecting HIV-PULSE's specificity for this subtype. Patient characteristics are described in Table 1 and Supplementary Table S1, for which all terms are explained in the glossary (Supplementary Table S2). Although selection and inclusion criteria may introduce some degree of bias, the study subset remains heterogeneous with respect to demographic and clinical characteristics, including sex at birth and mode of transmission. Overall, the participants included in this study are considered broadly representative of the larger 2000HIV cohort (Supplementary Table S3).

**Table 1:** Clinical characteristics of the study participants.

Characteristic	2000HIV *N* = *90*
	Median [IQR]
Age	54.0 [45.0–58.0]
BMI at baseline (kg/m^2^)	24.8 [22.7–27.1]
HIV duration (years)	13.7 [9.31–20.0]
Time to ART (days)	319 [41.5–866]
ART duration (years)	11.7 [7.29–15.4]
Viral load zenith (copies/mL)	125,000 [70,120–287,588]
CD4 nadir (10^9^cells/mL)	0.3 [0.2–0.4]
CD4 pre-ART (10^9^cells/mL)	0.3 [0.2–0.4]
CD8 pre-ART (10^9^cells/mL)	1.0 [0.7–1.5]
CD4/CD8 ratio pre-ART	0.3 [0.2–0.4]
CD4 latest (10^9^cells/mL)	0.9 [0.6–1.1]
CD8 latest (10^9^cells/mL)	0.8 [0.6–1.2]
Total HIV-1 DNA copy number (copies/10^6^ CD4 cells)	712 [404-1111]
Intact HIV-1 DNA copy number (Rainbow, copies/10^6^ CD4 cells)	32 [7–58.5]
	n (%)
Sex at birth	
Female	14 (15.6%)
Male	76 (84.4%)
Ethnicity	
Asian	2 (2.2%)
Black	7 (7.8%)
Hispanic	1 (1.1%)
Mixed	1 (1.1%)
White	79 (87.8%)
NRTI class	
No	0 (0.00%)
Yes	90 (100%)
NNRTI class	
No	45 (50.0%)
Yes	45 (50.0%)
PI class	
No	86 (95.6%)
Yes	4 (4.4%)
INSTI class	
No	48 (53.3%)
Yes	42 (46.7%)
Drug resistance mutations (based on clinical file)	
No	26 (28.9%)
Yes	8 (8.9%)
Unknown	56 (62.2%)
HLA-A∗02:01	
Present	36 (40.0%)
Absent	42 (46.7%)
Unknown	12 (13.3%)

Terms are explained in the glossary (Supplementary Table S2).

### Ethics

The Medical Ethical Review committee Nijmegen approved the 2000HIV study protocol (NL68056.091.81). All participants provided written consent before inclusion in the study.

### DNA isolation and HIV-1 DNA measurements

CD4 T cells were enriched by negative selection using the EasySep Human CD4+ T cell isolation kit on the RoboSep™-S (Stemcell Technologies) from thawed PBMC. gDNA was isolated via column extraction using the QIAamp DNA Mini kit on the Qiacube (Qiagen) with two elution steps of 50 μL. DNA concentrations were determined using 2 μL of the eluted DNA with the SpectraMax Quant AccuBlue HiRange dsDNA Assay Kit by using the SpectraMax i3x (Molecular Devices). Samples were stored at −20 °C prior to dPCR quantification. Total and intact HIV-1 copy number were determined by a multiplex dPCR assay (QIAcuity, Qiagen), as previously described by the dPCR Rainbow assay.^43^ The assay measured five different genomic regions, using the following cycling program: 2 min at 95 °C; 40 cycles (30 s at 94 °C, 1 min at 56 °C).^43^ Primers and probe sequences were acquired from Bruner et al.,^44^ Gaebler et al.,^45^ Yun et al.,^46^ and Yu et al.^47^ (Supplementary Table S4). Data was analysed using the Rainbow Shiny tool (https://digpcr.shinyapps.io/rainbow5_hiv_dna_qiacuity/). HIV-1 DNA levels were normalised by measuring the reference gene RPP30 in duplicate and reported per million CD4 T cells. Total HIV-1 copy number is based on RU5+ partitions and intact HIV-1 DNA is based on *psi*, *env*, *gag*, *pol* quadruple-positive partitions.

### HIV-PULSE

Near full-length proviral genomes were obtained by a modified version of the HIV Proviral UMI-mediated Long-read Sequencing (HIV-PULSE) assay.^38^ To enhance read quality, modifications were made to the pre-amplification enzyme, sequencing kits, and analysis software, resulting in an accuracy improvement from 99.91% to 99.95%. The modified protocol can be found here: https://doi.org/10.17504/protocols.io.8epv5rby4g1b/v2.^48^ The list of primers is included in Supplementary Table S4.

#### Pre-amplification

During the first PCR, HIV-1 proviral templates are pre-amplified using a high-fidelity enzyme and the outer primers of a nested HIV-1 primer set. For each individual, the assay was performed in six replicates containing each 500 ng gDNA (corresponding with ∼82,500 CD4 T cells), 1 μL of PrimeSTAR GXL DNA Polymerase (Takara Bio, #R050B), 0.2 μM of each primer (First PCR F, First PCR R), 4 μL of dNTP mixture, 10 μL of  5X PrimeSTAR GXL Buffer in a final volume of 50 μL. The following cycling conditions were used: 98 °C for 2 min; 6 cycles (10 s at 98 °C, 15 s at 65 °C, 10 min at 68 °C); 68 °C for 10 min. PCR products were cleaned using CleanPCR magnetic beads (CleanNA, #CPCR-0050) at a 1.0 × beads:sample ratio.

#### HIV-1 templates tagging

A second PCR using adapted primers was performed so that both ends of the pre-amplified proviral HIV-1 templates were tagged with a tailed UMI. The primers consisted of three parts: (i) an HIV-1 inner primer of the nested primer set to target the pre-amplified templates, (ii) a UMI with repetitive pattern of 12 random nucleotides and 6 degenerate nucleotides (Y/R) and (iii) a synthetic primer binding site for further amplification. Each PCR reaction contained all the cleaned pre-amplified product (30 μL), 2 μL of LongAmp Hot Start Taq DNA Polymerase (NEB, #M0534), 0.5 μM of each primer (Second PCR F UMI, Second PCR R UMI), 1.5 μL of 10 mM dNTPs (Promega, #C1141), 10 μL of 5 × LongAmp Taq Reaction Buffer in 50 μL. The following cycling conditions were used: 94 °C for 1 min 15 s; 2 cycles (94 °C for 30 s, 58 °C for 30 s, 65 °C for 10 min); 65 °C for 10 min. A custom buffer solution, based on the ‘SPRI size selection protocol for >1.5–2 kb DNA fragments’ protocol provided by Oxford Nanopore Technologies (ONT), was used to clean the tagged PCR products at a 0.9 × beads:sample ratio and eluted in 30 μL nuclease-free water.

#### Amplification of UMI-tagged proviruses

In order to have enough template input required for long-read sequencing, four consecutive PCR amplification rounds of each 10 cycles followed by a cleanup are performed. The primer set used in these amplification rounds binds to the synthetic binding site incorporated during the tagging stage. Each PCR reaction contained a mix consisting of 2 μL of LongAmp Hot Start Taq DNA Polymerase (NEB, #M0534), 0.5 μM of each primer (ncec_pcr_fw_v7, ncec_pcr_rv_v7), 1.5 μL of 10 mM dNTPs (Promega, #C1141), 10 μL of 5 × LongAmp Taq Reaction Buffer in 50 μL. During the first PCR amplification round all the cleaned tagging products from the previous step (30 μL) were used as template inputs, while only 10 μL of the cleaned product of the previous round were used during the second and third amplification rounds. The following cycling conditions were used: 94 °C for 1 min 15 s; 10 cycles (94 °C for 30 s, 60 °C for 30 s, 65 °C for 10 min); 65 °C for 10 min. For the fourth amplification round 20 μL of the cleaned product of the third round was used. The cycling conditions for the last PCR were: 94 °C for 1 min 15 s; 10 cycles (94 °C for 30 s, 61 °C for 30 s, 65 °C for 10 min); 65 °C for 10 min. PCR products were cleaned after each consecutive round using regular CleanPCR magnetic beads (CleanNA, #CPCR-0050) at a 1.0 × beads:sample ratio and eluted in 30 μL of nuclease-free water. During the fourth amplification round, a custom set of tailed primers were used to barcode the PCR products from the same participant with a specific, identical identifier. After the amplification rounds, the products were visualised on a 1% agarose gel and the DNA concentration was determined using a Spectramax i3X reader (Molecular Devices) with the Quant-iT PicoGreen dsDNA assay kit (ThermoFisher Scientific, #P7589).

#### ONT long-read sequencing

Samples were multiplexed using the Native Barcoding Kit 24 V14 kit (ONT, #SQK-NBD114-24) following this approach: each PCR replicate was assigned a unique ONT barcode and included into an equimolar pool of PCR products from different participants. This enabled the later identification of reads based on the ONT barcode corresponding to the PCR replicate and the participant-specific identifier added during the final PCR round. For the library preparation, the Native Barcoding Kit 24 V14 (ONT, #SQK-NBD114-24) was used according to the manufacturer's instructions except for the end-prep. During this step, 48 μL, 3.5 μL and 3 μL was used of the 200 fmol amplicon DNA pool, Ultra II End-prep reaction buffer and Ultra II End-prep enzyme mix (NEB, #E7546), respectively.

Samples were sequenced on a MinION ONT device using MinION R10.4.1 flow cells (ONT, #FLO-MIN114) and the MinKNOW v.23.04.5 software followed by basecalling at super accuracy mode and demultiplexing with Guppy v6.4.2 and v6.5.7.

### Bioinformatic analysis of long-read data

As previously described, the long-read data was analysed using a customised longread UMI HIV pipeline and HIV-PULSE workflow (https://github.com/HCRCugent/longread_umi_hiv).^38^ In brief, the following consecutive steps were followed: (i) trimming and filtering of the long-read sequencing data using Porechop (v.0.2.4, https://github.com/rrwick/Porechop), Filtlong (v.0.2.0, https://github.com/rrwick/Filtlong) and cutadapt (v.2.7)^49^; (ii) extraction of UMI reference sequences using cutadapt (v.2.7) and usearch^50^; (iii) binning of reads to UMI combinations using bwa (v.0.7.17) and samtools (v.1.11); (iv) generation of bin centroid sequences using usearch and minimap2 (v.2.17)^51^ and (v) polishing of bin centroid data by multiple rounds of racon (v.1.4.20)^52^ and a final round of Medaka (v.1.4.3, https://github.com/nanoporetech/medaka). The custom HIV-PULSE specific workflow allowed to correct for pre-amplification errors, improve final bin accuracy and evaluate clonality among PCR replicates.

### HIV-1 genome classification

NFL proviral genome classification was performed using the publicly available ‘Gene Cutter’ and ‘Hypermut’ webtools from the Los Alamos National Laboratory HIV sequence database (https://www.hiv.lanl.gov). Proviral genomes were classified in the following sequential order: (i) ‘Inversion’: presence of internal sequence inversion, defined as region of reverse complementarity. (ii) ‘Large internal deletion’: internal sequence deletion of >600 bp. (iii) ‘Hypermutated’: APOBEC-3G/3F-induced hypermutation. For each participant, a consensus sequence was generated from the remaining genomes and used as the reference for a new alignment. This alignment was then analysed using the ‘Hypermut’ webtool, with proviruses scoring P < 0.05 considered to be hypermutated. (iv) ‘PSI/MSD defect’: proviruses containing a deletion >7 bp found in any of the four stem loops of the *psi* region (SL1 (HXB2: 691–734), SL2 (HXB2: 736–754), SL3 (HXB2: 766–779) and SL4 (HXB2: 790–810)). This also includes the absence and/or point-mutation of GT dinucleotide at both the MSD site and the cryptic donor site. Proviruses with a deletion covering PSI/MSD that extended into the *gag* gene, thereby removing the *gag* AUG start codon, were also classified into this category. (v) ‘Premature stop-codon/frameshift’: premature stop-codon or frameshift caused by mutation and/or sequence insertion/deletion in the essential genes *gag*, *pol,* or *env*. Proviruses with insertion/deletion >49 nt in *gag*, insertion/deletion >49 nt in *pol*, or insertion/deletion >99 nt in *env* were also classified into this category. (vi) ‘Intact’: proviruses that displayed none of the above defects were classified into this category.

### Partial *env* sequencing

The env region of HIV was amplified using a nested PCR strategy, resulting in a ∼1.1 kb fragment. The outer PCR contained 2 μL of gDNA, 2 μL 5X PrimeSTAR GXL Buffer, 250 nM of each primer, 200 μM dNTPs and 1.25U PrimeSTAR GXL DNA polymerase (Takara Bio, #R050B) in a final volume of 10 μL. The nested inner PCR reaction contained 2 μL of the first-round product, 8 μL 5X PrimeSTAR GXL buffer, 250 nM of each primer, 200 μM dNTPs, and 1.25 U PrimeSTAR GXL in a 40 μL final reaction volume. The following cycling conditions were used for both PCRs: 98 °C for 2 min; 30 cycles (10 s at 98 °C, 15 s at 55 °C, 1 min at 68 °C); 68 °C for 5 min. For each participant, the PCR reactions were performed in triplicate. The resulting *env* amplicons were Sanger sequenced using four primers to ensure complete coverage. All primers are listed in Supplementary Table S4. Following quality assessment, reads from all primers were assembled independently for each replicate to generate *env* consensus sequences. These high-quality consensus sequences were used for subtyping and tropism determination. The HIV subtypes were determined using the REGA subtyping tool (v3.47)^53^ as a primary method. Cross-validation of the results was performed with COMET^54^ and Geno2pheno virus detection and subtyping tool,^55^ if the subtype assignment support was below 70%. Co-receptor usage was assessed by the Geno2pheno coreceptor tool using the *env* V3 loop sequences with a false discovery rate (FDR) of 10%.^56^ If all replicates showed the same tropism, individuals were assigned to the R5 or X4 group, whereas differing results among replicates were classified as mixed tropism.

### Testing of tropism, subtyping and diversity of long-read data

Proviral sequences were aligned against the HXB2 reference sequence using MAFFT (v7.520).^57^ Proviral sequences with inversions or hypermutations were excluded from further analyses. Subtyping predictions were done using Geno2pheno virus detection and subtyping tool.^55^ For tropism prediction, proviral sequences with full coverage of V3 region in *env* were retained. The prediction was performed via Geno2pheno coreceptor v2.5, a widely used and clinically validated genotypic tropism prediction tool,^34^ applying a 10% FDR.^56^ Multiple sequence alignment of HIV-PULSE sequences was performed using MAFFT (v7.520).^57^ Pairwise diversity among proviral sequences was calculated using Analysis of Phylogenetics and Evolution (v5.8-1, https://github.com/emmanuelparadis/ape). Gaps among the proviral sequences were handled in a pairwise manner. Phylogenetic trees were reconstructed with IQ-TREE2,^58^ using the built-in model finder and 1000 bootstraps. Visualisation and annotation of trees was done by R (v.4.3.3), ggplot2 (v.3.5.1) and ggtree (v.3.10.1).^59^ Recombination analysis was performed by the Recombination Detection Program 5 (RDP5).^60^

### Drug resistance mutations analysis

Drug resistance-associated mutations were identified using the Stanford University HIV Drug Resistance Database (HIVDB) algorithm (v9.8).^61^ Pre-ART plasma *pol* sequences spanning the protease and reverse transcriptase were available for 20 individuals. *Pol* sequences retrieved from HIV-PULSE, excluding those containing inversions and hypermutations, were analysed alongside the available pre-ART plasma sequences as input to the HIVDB algorithm.

### Sensitivity to broadly neutralising antibodies and Gag-targeted therapies

Proviral sequences were translated into amino acids for downstream analyses. The *env*-derived amino acids were used to predict sensitivity to bNAbs through the bNAb-Resistance Predictor (bNAb-ReP) algorithm,^62^ with a probability value ≥ 0.50 corresponding to neutralisation sensitivity. To assess susceptibility to the immune-mobilising monoclonal T cell receptor IMC-M113V, the Gag^77–85^ region of each proviral sequence was compared against one of the eight IMC-M113V target epitopes.^32^ Available participant-specific HLA class I data were incorporated to assess potential recognition of the IMC-M113V Gag^77–85^ epitope.

### Statistics

Statistical analyses were performed using R software (v.4.3.3). Univariate analyses were conducted to assess associations between the average pairwise distance and clinical parameters using a Spearman test. The normality of the data was tested using Shapiro–Wilk test. The p-values were corrected for multiple comparisons using the Benjamini-Hochberg (BH) method with a significance level of 0.05. Variables with a p-value <0.2 in the univariate analysis were considered for inclusion in the multivariate analysis. A multivariable linear regression model was constructed to identify independent predictors of pairwise diversity. Model selection was performed using forward selection to optimise model fit and parsimony. Multicollinearity among predictors was evaluated using correlograms. Based on the final multivariable model, partial Spearman correlation coefficients were computed to assess adjusted associations between pairwise diversity and variables.

### Role of funders

This research is part of the 2000HIV study, which is supported by ViiV Healthcare. ViiV Healthcare retained the right to review the content of the manuscript. However, they did not have any role in data handling, statistical analyses, writing or final interpretation of the data.

### Data deposition and materials sharing

HIV-1 sequencing data supporting the findings of this study have been deposited in GenBank with the accession code: PX947546-PX951409. The scripts used in this study are publicly available on GitHub: https://github.com/HCRCugent/longread_umi_hiv. The GitHub page provides a description of the key operations and instructions on how to install and run the code.

## Results

### Participant and reservoir baseline characteristics

We performed an in-depth characterisation of the HIV-1 proviral reservoir in a subset of 90 ART-suppressed people living with HIV from the 2000HIV study.^40^ Participants were selected based on: (i) sample availability; (ii) subtype B determination through *env* sequencing; and (iii) >3 HIV-1 cp/μL DNA input per HIV-PULSE replicate (Supplementary Fig. S1A). Although selection was based on reservoir size, the resulting subset centres around the interquartile range, ensuring representation of the whole 2000HIV cohort rather than outliers (Supplementary Fig. S1B). The selected subset consisted predominantly of white males sampled with a median of 13.7 years since HIV-1 diagnosis. General participant characteristics of the subset are described in Table 1 and Supplementary Table S1.

### HIV-PULSE allows high-throughput classification of viral sequences

**Fig. 1:**
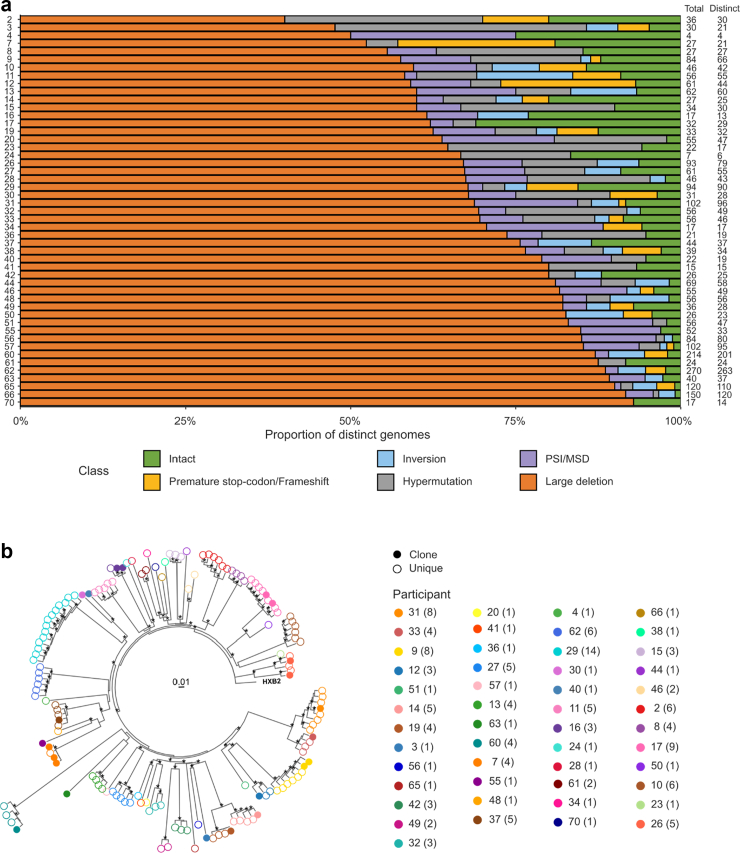
**Proviral reservoir characterisation and classification.** Assessment of the proviral reservoir via HIV-PULSE in participants with intact proviruses. a) Proportions of different proviral classes among distinct proviral genomes in each participant with at least one intact provirus. Total and distinct provirus counts are displayed to the right of each bar. b) Phylogenetic tree of distinct intact proviral genomes (n = 150). Each participant (n = 49) is indicated by a unique colour, with the order corresponding clockwise to the legend. Numbers in brackets indicate the total number of intact sequences per participant. Filled dots represent sequences detected across multiple PCR replicates, whereas empty dots indicate sequences are found in a single PCR replicate (unique). Asterisks indicate nodes with bootstrap support >75%.

The HIV-PULSE amplification strategy was applied to genomic DNA (gDNA) from 90 selected participants of the 2000HIV-cohort. Seven participants were excluded for Nanopore sequencing due to insufficient DNA yields after amplification. Among the remaining 83 participants, the mean PCR success rate was 91.4%, defined as the proportion of successful PCR replicates for sequencing. These 83 individuals, for whom downstream analyses were performed, are hereafter referred to as “all participants”. To examine the reservoir composition, proviral sequences were classified into intact or defective proviruses. A total of 4507 proviruses were identified across all participants, with a median of 44 proviruses per participant (IQR: 26–68; Supplementary Table S5). When considering only unique proviruses, 4040 distinct proviruses were identified. The majority were defective consisting of 81.5% large deletion, 5.1% packaging signal and/or major splice donor (PSI/MSD) defect, 4.3% hypermutation, 3.4% inversion and 2.0% premature stop-codon or frameshift (Fig. 1A, Supplementary Fig. S2, Supplementary Table S5). On average, 3.7% of the unique proviruses is considered putatively intact, with individual proportions ranging from 0 to 31.0%.

### Genetic redundancy and clonality of detected proviruses

Next, we identified genetically identical HIV-PULSE sequences found across multiple replicates, which are indicative of clonality. Because a pre-amplification step precedes unique molecular identifier (UMI) tagging, identical sequences arising within a single replicate cannot be distinguished from repeated amplification of the same template. Consequently, the clonality reported here represents an underestimation of the true clonal size. Clonal sequences were identified among both defective and intact proviruses. Intact proviruses were identified in 49 participants, of which 17 showed indications of clonality, with genetically identical intact sequences found across multiple replicates (15.3% of all intact sequences). A maximum-likelihood phylogenetic tree of the intact proviral sequences represents the diversity of intact proviruses across participants (Fig. 1B). Whereas only a single intact clone was detected in 12 individuals, multiple distinct intact clones were detected in five individuals.

### HIV-PULSE identifies minority variants of mixed-tropic proviruses

**Fig. 2:**
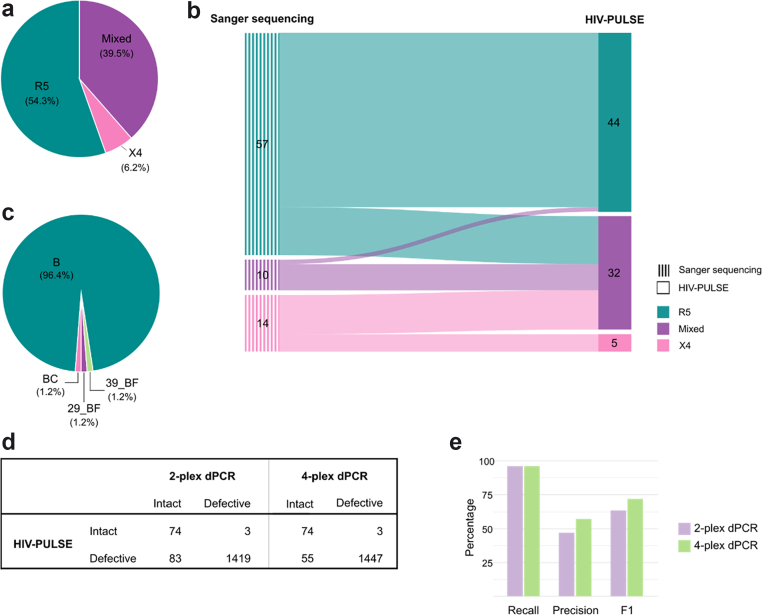
**Characterisation of proviral tropism, subtype diversity, and reservoir quantification design using HIV-PULSE.** a) Proportion of predicted coreceptor usage among 81 participants. b) Comparison of tropism predictions between conventional *env* Sanger sequencing and HIV-PULSE. Sankey diagram shows the number of participants predicted to harbour X4-, R5-, or mixed-tropic proviruses by each method. c) Proportion of predicted subtyping across all individuals (n = 83). d) Table summarising the HIV-PULSE validation of quantitative reservoir assays. The number of intact and defective proviral genomes based on HIV-PULSE sequences and the corresponding intact and defective proviruses by two regions (psi and *env*) and four regions (psi, *gag*, *pol* and *env*) in the Rainbow DNA assay are shown. e) Comparison of performance metrics for 2-plex and 4-plex dPCR relative to HIV-PULSE sequences. The F1 score represents the harmonic mean of precision and recall, and provides a balanced measure of classification performance.

Using the *env* region of each provirus, with proviruses containing inversions or hypermutations excluded, we predicted coreceptor usage to determine whether individual proviruses were CCR5- or CXCR4-tropic. Across the cohort, viral tropism could be determined for 81 individuals based on multiple proviral sequences per individual (ranging from 2 to 96 proviruses analysed). Among these, proviruses from 44 participants were predicted to use CCR5 exclusively (54.3%) and five CXCR4 exclusively (6.2%), while 32 individuals harboured both R5- and X4-tropic proviruses referred to as mixed-tropic (39.5%), showing within-host coexistence of the two coreceptor tropisms (Fig. 2A). When compared with tropism determined by *env* Sanger sequencing^34^ from the same samples, HIV-PULSE revealed a greater proportion of mixed-tropic individuals (39.5% vs 12.3%; Fig. 2B), suggesting increased sensitivity for detecting minority variants. By generating individual proviral genomes rather than consensus-based *env* sequences, HIV-PULSE reveals minority variants with potential implications for the use of R5-targeted therapies.

### HIV-PULSE enables refined subtype determination

Because HIV-PULSE sequences multiple HIV-1 proviral genomes for each participant, we performed subtyping analysis using Geno2Pheno to assess potential recombinational forms across the cohort. Participants had originally been classified as subtype B based on *env* sequencing. Subtyping of HIV-PULSE-derived sequences confirmed subtype B in 96.4% of participants, demonstrating overall concordance with conventional *env*-based subtyping (Fig. 2C). However, in three individuals, HIV-PULSE revealed proviruses predicted to belong to recombinant circulating forms (BC, 29_BF and 39_BF), revealing previously undetected intersubtype recombination. These findings highlight that NFL proviral sequencing provides a more accurate assessment of viral subtype determination than *env*-only sequencing.

### HIV-PULSE identifies critical target sites for reservoir quantification assays

To evaluate the precision of intact provirus quantification, which depends on primers/probe performance, we performed an in silico validation using HIV-PULSE-derived NFL proviral genomes in a subset of 25 participants, yielding 1579 sequences, of which 77 were classified as putatively intact. The Rainbow HIV-1 DNA digital PCR (dPCR) assay targets four genomic regions (psi, *gag*, *pol* and *env*). We mapped each primers/probe set to the corresponding proviral sequences and assessed potential mismatches, defining a maximum of three mismatches per primer or probe and a total of five per primers/probe set.^63^

Our analysis predicted that 55 defective proviruses would be misclassified as intact using the 4-plex Rainbow assay (Fig. 2D), corresponding to a precision of 57.4% (Fig. 2E). When only the psi and *env* regions were considered, the number of predicted misclassifications increased to 83 and precision decreased to 47.1%, highlighting the added value of targeting multiple regions for accurate assessment of intactness. In three cases, intact proviruses exceeded the allowed mismatch threshold in the psi probe region and were therefore predicted to be incorrectly classified as defective. In one participant, the Rainbow HIV-1 dPCR indicated a psi-region dropout, whereas in silico mapping suggested potential primers/probe binding. However, nearly all sequences exhibited three mismatches within the probe region, suggesting that cumulative mismatches may reduce probe efficiency and lead to signal loss. Together, these findings demonstrate the use of HIV-PULSE for validating and refining primer/probe design in quantitative reservoir assays.^43^

### HIV-PULSE reveals distinct viral lineages consistent with multiple infections

**Fig. 3:**
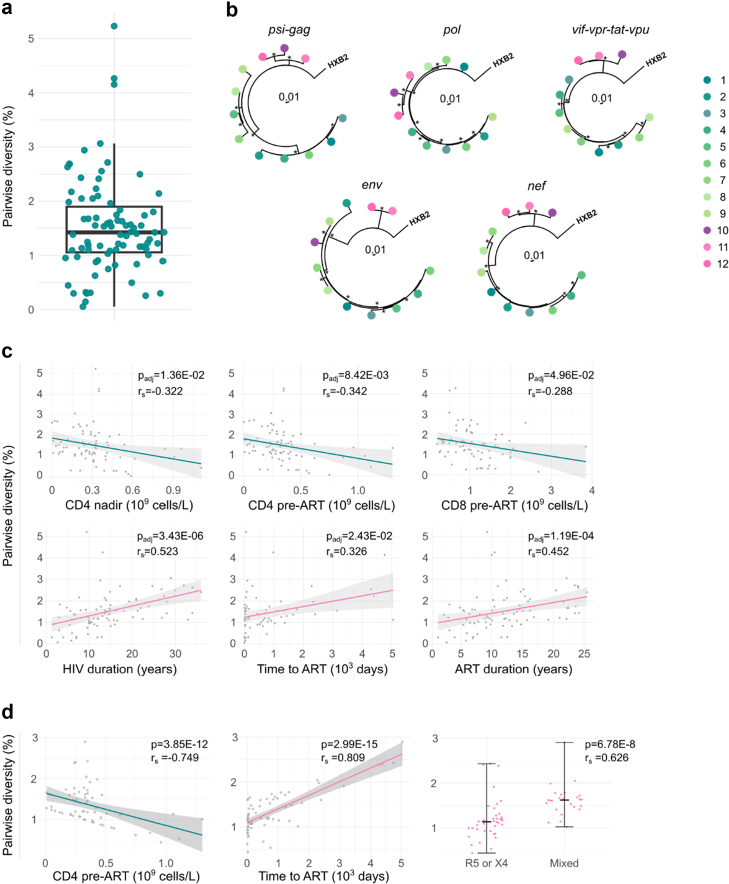
**Proviral diversity of the reservoir in relation to clinical characteristics.** a) Distribution of the mean pairwise genetic diversity among proviral sequences in 83 participants. b) Phylogenetic trees including intact and PSI/MSD defective sequences from participant 26 across five genomic regions. Each unique provirus is shown as a different coloured dot. Asterisks indicate nodes with bootstrap support >75%. c) Univariate correlations between pairwise diversity and clinical characteristics (Spearman correlation, Table 2). d) Partial spearman correlations derived from a multivariable model including time to cART, CD4 pre-cART and mixed tropism. Correlation coefficient and p values are shown. Terms are explained in the glossary (Supplementary Table S2).

The genetic proviral diversity was evaluated within participants by calculating the average pairwise distance including both intact and defective proviruses (specifically large deletions, PSI/MSD defective, and those containing premature stop-codons or frameshifts). The results revealed a median pairwise diversity of 1.4% [0.05–5.2%], with three participants exhibiting proviral diversity exceeding 4% (Fig. 3A). In one of these individuals, the observed diversity of 4.3% raised the possibility of multiple infections. Phylogenetic trees were reconstructed from NFL sequences (including both intact and PSI/MSD defective sequences from this participant) across five different HIV-1 genomic regions (Fig. 3B). Three proviruses cluster separately from the others in all regions, except for *env* where only two out of three proviruses cluster. The other provirus suggests a recombination between the two viral strains. Recombination and breakpoints were confirmed using RDP5 (Supplementary Fig. S3). In the other two participants with >4% diversity, either only a limited number of sequences were available or sequences were deleted, precluding similar phylogenetic analyses.

### Proviral diversity reflects immune status, treatment initiation timing and viral tropism

To explore the determinants of proviral diversity, we correlated pairwise diversity with available clinical and viral reservoir data of the cohort (Table 2). Initial univariate correlation analysis showed significant positive correlations between the average pairwise diversity and HIV-1 duration, time to ART (from HIV-1 diagnosis to treatment initiation) and ART duration (p_adj_ = 3.4E-06; 2.4E-02; 1.2E-04), while a negative correlation was observed with CD4 nadir, CD4 pre-ART and CD8 pre-ART (p_adj_ = 1.4E-02; 8.4E-03; 4.9E-02; Fig. 3C, Supplementary Fig. S4). A multivariate model analysis was performed by excluding non-significant variables and mitigating collinearity among predictor variables by retaining one variable from each correlated pair (Supplementary Fig. S4). In the subsequent multivariate linear regression model, time to ART, CD4 pre-ART and mixed tropism remained significant predictors of proviral diversity (p < 0.05). Using the fitted data of the multivariate model, we calculated partial spearman correlations. PLWH having a longer time to treatment initiation and mixed tropism exhibited a higher proviral diversity (p = 2.9E-15; 3.9E-08), whereas a higher pre-ART CD4 cell count demonstrated a reduced diversity (p = 2.7E-12; Fig. 3D).

**Table 2:** Correlation assessment of pairwise diversity with clinical and viral reservoir parameters.

	Pairwise diversity		
	Rho	p	p_adj_
Age	0.13	0.25	0.39
BMI at baseline (kg/m^2^)	0.09	0.41	0.57
Year HIV diagnosis	−0.53	<0.001	<0.001
CD4 nadir (10^9^cells/mL)	−0.32	0.00	0.01
CD4 pre-ART (10^9^cells/mL)	−0.34	0.00	0.01
CD8 pre-ART (10^9^cells/mL)	−0.29	0.02	0.05
CD4/CD8 ratio pre-ART	−0.05	0.71	0.82
CD4 latest (10^9^cells/mL)	−0.06	0.56	0.72
CD8 latest (10^9^cells/mL)	0.04	0.78	0.86
CD4/CD8 ratio latest	0.00	0.98	0.99
Viral load zenith (copies/mL)	−0.16	0.17	0.29
HIV duration (years)	0.52	<0.001	<0.001
Year start ART	−0.44	<0.001	<0.001
ART duration (years)	0.45	<0.001	<0.001
Time to ART (days)	0.33	0.01	0.02
Total HIV-1 DNA copy number (copies/10^6^ CD4 cells)	0.21	0.06	0.13
IPDA - intact HIV-1 DNA copy number (copies/10^6^ CD4 cells)	−0.01	0.96	0.99
Rainbow - intact HIV-1 DNA copy number (copies/10^6^ CD4 cells)	0.00	0.97	0.99
Center	−0.07	0.55	0.93
Cohort	−0.01	0.96	0.99
Sex at birth	0.21	0.06	0.42
Ethnicity	0.10	0.37	0.93
Ethnicity_white	−0.10	0.35	0.93
Ethnicity_black	0.11	0.32	0.92
Ethnicity_Asian	0.04	0.72	0.94
Ethnicity_Hispanic	0.06	0.56	0.93
Ethnicity_mixed	−0.07	0.51	0.93
Current smoking	0.19	0.10	0.63
Smoking ever	−0.04	0.76	0.94
Risk behaviour	0.13	0.24	0.88
Acute HIV	−0.42	0.01	0.09
Drug resistance mutations (based on clinical file)	0.03	0.89	0.96
Residual viraemia	0.07	0.51	0.93
Blips	−0.24	0.35	0.93
Viraemia	0.00	1.00	1.00
Rapid progressor	−0.05	0.72	0.94
Immunological non-responder	0.16	0.18	0.85
Controller	0.09	0.43	0.93
Subtype	−0.05	0.67	0.94
Tropism	0.27	0.01	0.18
Tropism_X4	−0.08	0.46	0.94
Tropsim_R5	−0.23	0.04	0.34
Tropism_Mixed	0.27	0.01	0.17
HiViReT	0.15	0.19	0.85
Early ART (30 days)	−0.21	0.09	0.58
Early ART (6 months)	−0.18	0.14	0.78

Correlations between the pairwise diversity and available clinical and viral reservoir data were determined using Spearman correlation. Multiple comparison correction was performed using the Benjamini-Hochberg method.

### Characterisation of archived drug resistance in the proviral reservoir

#### HIV PULSE drug resistant mutations belonging to intact and defective viral sequences

**Fig. 4:**
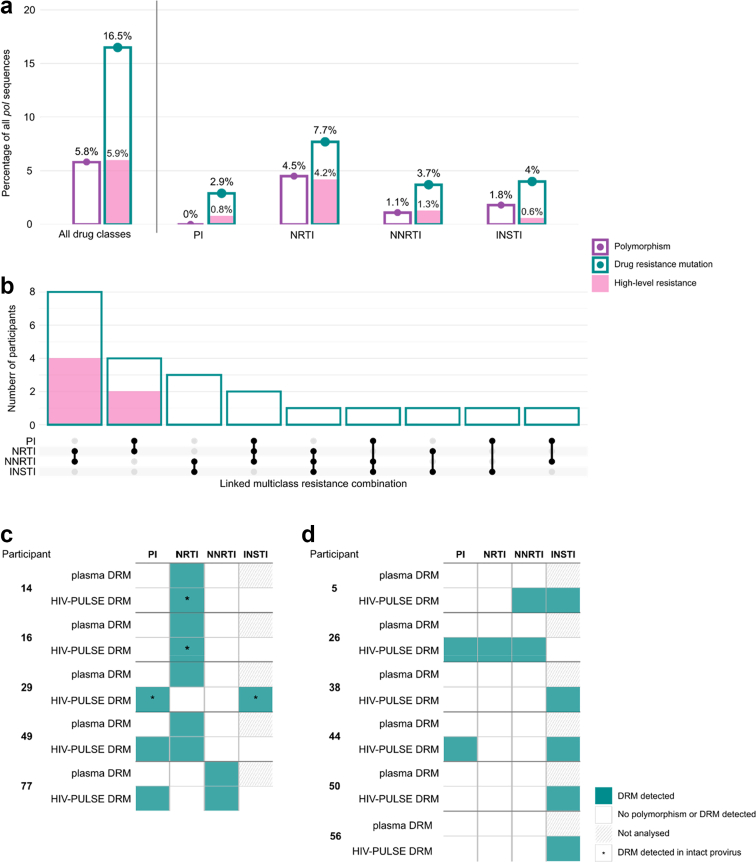
**Prevalence and patterns of HIV-1 drug resistance mutations.** Drug resistance was assessed according to the estimated levels of resistance by the Stanford HIV Drug Resistance Database. a) Percentage of polymorphisms, drug resistance mutations and high-level resistance mutations across all analysed *pol* sequences by HIV-PULSE (n = 2235). b) Number of participants harbouring drug resistance mutations affecting multiple drug classes in a single provirus. High-level resistance against multiple drug classes is highlighted in pink. c) Schematic overview of drug resistance mutations identified in both pre-ART plasma and on-ART gDNA. d) Schematic overview of drug resistance mutations identified in gDNA that were not detected in pre-ART plasma. Integrase resistance testing was not available for plasma-derived sequences. Terms are explained in the glossary (Supplementary Table S2).

To assess the prevalence and distribution of DRMs in the cohort, we analysed the complete *pol* region for resistance-associated mutations across all participants. Mutations were categorised as either polymorphisms (mutations not associated with reduced susceptibility to antiretrovirals) or DRMs (mutations conferring potential low- to high-level resistance), according to the Stanford HIV Drug Resistance Database. In total, 2235 *pol* sequences from unique intact and defective proviruses were examined. Of these, 497 sequences from 60 participants harboured at least one mutation, encompassing polymorphisms (n = 129), DRMs (n = 47), or both (n = 321) (Fig. 4A, Supplementary Table S6). At the sequence level, DRMs were most associated with nucleoside reverse transcriptase inhibitors (NRTIs, 7.7% of all sequences tested), whereas at participant level, DRMs were most frequently associated with integrase strand inhibitors (INSTIs, 34.9% of all participants). This difference likely reflects a broader distribution of INSTI-associated DRMs across the cohort, in contrast to a clustering of multiple NRTI-associated DRMs within sequences from a smaller subset of individuals. By mapping each DRM to its corresponding proviral context, we found that 9.0% of all DRMs (n = 33) occurred within intact proviruses, spanning NRTIs (n = 20), non-nucleoside reverse transcriptase inhibitors (NNRTIs, n = 9), INSTIs (n = 3) and protease inhibitors (PIs, n = 2).

High-level resistance (HLR) to at least one antiretroviral drug was predicted in 132 *pol* sequences across 39 participants, with a distribution across drug classes similar to that of DRMs overall (Fig. 4A). At both sequence and participant level, HLR was most commonly associated with NRTIs. The most frequent HLR among individuals was observed against emtricitabine (FTC) and lamivudine (3 TC), driven by one of two key mutations: M184I or M184V. When considering the proviral context, HLR was predominantly detected in defective proviruses, while only 6.1% of HLR-containing sequences were classified as intact. Across participants, the distribution of HLR between intact and defective proviruses varied between individuals: 33 showed HLR exclusively in defective proviruses, four in both types, and two only in intact proviruses.

#### HIV-PULSE identifies linked and unlinked multiclass resistance

To examine the co-occurrence of resistance mutations, we leveraged the multiple proviruses detected with HIV-PULSE in each participant by analysing individual proviruses for resistance spanning multiple drugs classes. Among participants with at least one DRM, 32 participants exhibited multiclass resistance. In 17 individuals, DRMs to multiple drug classes were linked on a single proviral sequence (Fig. 4B). In the remaining participants, multiclass resistance was unlinked and distributed across distinct proviruses. Supplementary Fig. S5 provides a schematic of these configurations. The most frequent linked and unlinked multiclass resistance was dual resistance to NRTIs and NNRTIs. Four-class resistance was only observed as unlinked across proviruses. Notably, 13 individuals exhibited multiclass HLR, which was unlinked in seven cases and linked in six cases. To further characterise the nature of multiclass resistance, we analysed these variants at the level of individual proviral genomes. The most frequent mutation patterns involved the NRTI mutation M184V in combination with either the PI mutation L90M or the NNRTI mutation G190A, conferring HLR to FTC and 3TC, together with dapivirine (DPV), efavirenz (EFV), nelfinavir (NFV), or nevirapine (NVP), respectively. Most multiclass-resistant sequences were classified as large deletion (n = 26). There was one intact proviral genome harbouring resistance against both NRTI (K65R) and NNRTI (E138A), leading to high-level resistance against NRTIs stavudine (D4T) and didanosine (DDI). These findings highlight the complexity of the proviral reservoir, with both intragenomic and intergenomic resistance patterns coexisting within the same individual.

#### HIV-PULSE maps the proviral DRM landscape and concordance with pre-ART plasma sequences

Finally, we compared proviral DRMs with historical pre-ART plasma sequences. For 20 participants, pre-ART plasma sequences covering the protease and reverse transcriptase region were available. DRMs were found in plasma of five individuals. Over time, these DRMs were retained in gDNA in three of five cases, and in two of these individuals the same DRMs were identified within intact proviruses (Fig. 4C, Table 3). Participant 29 had a DRM against NRTI in plasma, whereas the proviral sequences identified DRMs against PI and INSTI. In participant 49, the specific DRMs differed between timepoints (M41L pre-ART vs D67N in HIV-PULSE), yet both belong to the same class of thymidine analogue mutations. HIV-PULSE further detected additional DRMs beyond those detected in the plasma screening in nine individuals (Fig. 4C and D, Table 3).

**Table 3:** Presence of drug resistance mutations at the pre-ART plasma sequencing and on-ART HIV-PULSE timepoint.

Participant	Drug class	Pre-ART plasma DRM	HIV-PULSE DRM
14	NRTI	T215S	T215S^a^
16	NRTI	M41L,S68G,L210W,T215D	M41L,S68G,L210W,T215D^a^
29	PI	–	I50L^a^
	NRTI	M41L,S68G,L210W,T215D	–
	INSTI	–	E138A^a^
49	PI	–	G48V
	NRTI	M41L	D67N
77	PI	–	D30N,M46I
	NNRTI	E138A	E138A
5	NNRTI	–	G190E
	INSTI	–	S147G,E157Q
26	PI	–	M46I,G73S
	NRTI	–	M184I
	NNRTI	–	E138K,M230I
38	INSTI	–	V151A
44	PI	–	D30N,M46I
	INSTI	–	V75A,E138K
50	INSTI	–	G140C
56	INSTI	–	Q146L

Pre-ART plasma DRM compared to the DRM found at the on-ART HIV-PULSE timepoint. DRM, drug resistance mutation; PI, protease inhibitor; NRTI, nucleoside reverse transcriptase inhibitor; NNRTI, non-nucleoside reverse transcriptase inhibitor; INSTI, integrase strand inhibitor.

a DRM detected on intact provirus using HIV-PULSE.

### Sequence-based predictions reveal heterogeneous sensitivity to bNAbs

**Fig. 5:**
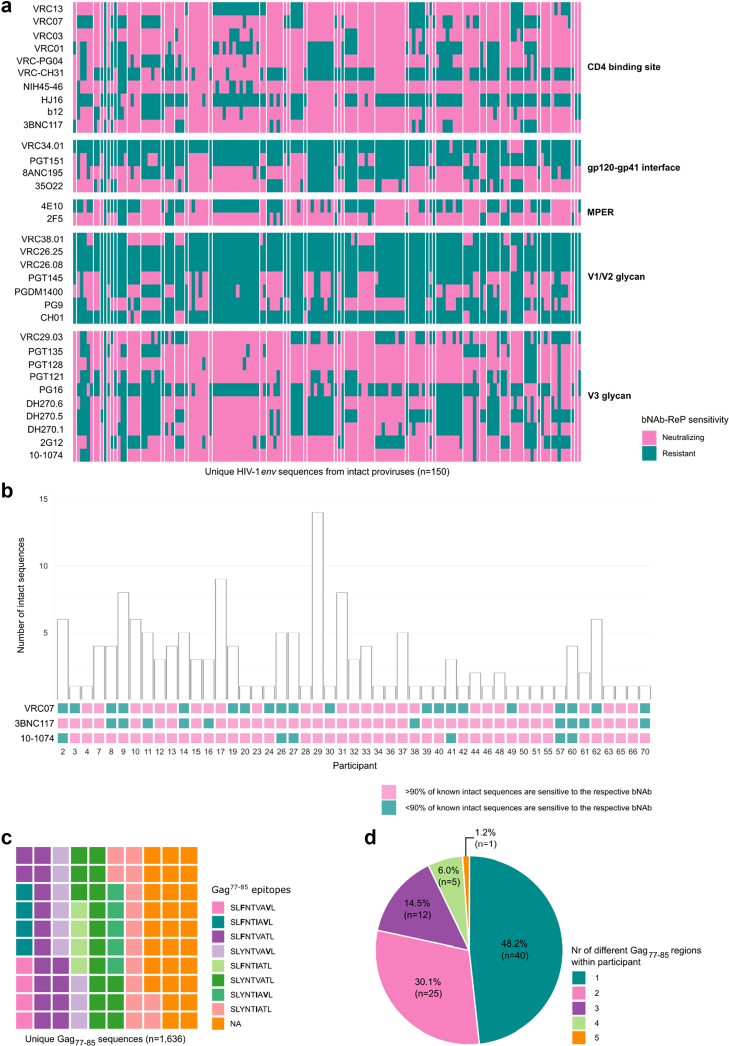
**In silico sensitivity prediction against immune-based therapies.** Proviral sequences were analysed in silico to predict susceptibility to novel immune-based therapies. a) Predicted resistance to bNAbs based on bNAb-ReP applied to HIV-1 *env* proviral sequences from intact proviruses (n = 150). Sequences from the same individual are grouped together and separated by a white space. Predictions are grouped by bNAb binding site. b) Heatmap showing the predicted eligibility of individuals for bNAbs currently or previously evaluated in clinical trials (3BNC117, 10–1074 and VRC07) based on intact proviruses. c) Waffle plot representing the distribution of Gag^77–85^ epitope variants in the cohort. The most common escape mutations are highlighted compared to the “cognate” sequence, SLYNTVATL. Results are shown for 1636 unique sequences containing this Gag^77–85^ region. d) Pie chart representing the within participant variation of the Gag^77–85^ region. Terms are explained in the glossary (Supplementary Table S2). MPER, membrane-proximal external region.

The detection of multiple NFL proviral sequences by HIV-PULSE within individuals allowed us to in silico predict sensitivity against emerging immune-based therapies like bNAbs and ImmTaVs. Using the *env* region of 150 distinct intact proviruses across 49 individuals, we performed in silico sensitivity predictions to a panel of 33 bNAbs (Fig. 5A). On average, 55.5% of the intact proviruses were predicted to be neutralised by at least one bNAb. Consistent with their recognition of a structurally conserved epitope, bNAbs directed against the CD4 binding site demonstrated the highest predicted efficacy, with an average of 69.5% [28.7–92.7%] sequences classified as sensitive. In contrast, V1–V2 apex-directed bNAbs were predicted to neutralise only 22.4% of the intact sequences. To explore differences in bNAb sensitivity between intact and defective proviruses, we extended the predictive analysis to 2612 distinct defective proviral sequences (Supplementary Fig. S6). These included sequences with defects in PSI/MSD, large deletions and a premature stop-codon, or frameshift. Overall, defective proviruses were more likely to show resistance to bNAbs compared to intact proviruses, with only 27.8% predicted to be neutralised by at least one bNAb (Supplementary Table S7). However, this discrepancy is mainly driven by proviruses with large deletions, i.e. when the *env* region is partially deleted. We observed that the PSI/MSD-defective proviruses exhibited a neutralisation pattern similar to that of intact proviruses.

Given that several bNAbs (3BNC117, 10-1074 and VRC07) are under clinical evaluation in studies such as eClear, Titan and RIO,^64^, ^65^, ^66^ we assessed potential participant eligibility based on HIV-PULSE-derived proviral sequences. Across all participants, 77 individuals harboured proviruses predicted to be sensitive to 3BNC117, 59 to VRC07, and 80 to the combination 3BNC117 plus 10–1074, corresponding to 92.8%, 71.1%, and 96.4% of participants, respectively. Among individuals with at least one intact provirus (n = 49), applying the >90% predicted sensitivity threshold previously used in clinical studies^64^ predicted 39 individuals eligible for trials involving 3BNC117 (47.0% of all participants), 48 for its combination with 10–1074 (57.8%), and 29 for those using VRC07 (34.9%) (Fig. 5B).

### HIV-PULSE reveals Gag^77–85^ polymorphism in the IMC-M113V target region

As alternative immune-based therapeutic strategies emerge, conserved and immunogenic regions like Gag have come into focus. IMC-M113V is a T cell receptor bispecific immunotherapy designed to redirect cytotoxic T cells towards HIV-1 infected cells presenting the Gag^77–85^ epitope in the context of HLA-A∗02:01. To evaluate the potential responsiveness of participants to this therapy, we performed in silico predictions of IMC-M113V sensitivity for the Gag^77–85^ region. A total of 1636 unique sequences containing this region were analysed. Among these, 72.1% matched one of the eight previously characterised Gag^77–85^ variants with published relative half-maximal effective concentration (EC_50_) values^32^ (Fig. 5C, Supplementary Tables S8 and S9), corresponding to 72 participants. Intra-individual variation within this epitope was observed in 43 participants (51.8% of all participants), including 22 individuals harbouring both known and unknown variants (Fig. 5D).

Because IMC-M113V targets the HLA-A∗02:01-restricted Gag^77–85^ epitope, we assessed HLA-typing for all individuals. 43.4% of all participant were HLA-A∗02:01 positive (n = 36), either homozygous (10), or heterozygous (26). Among these, 23 carried only Gag^77–85^ variants recognised by IMC-M113V, whereas 11 carried at least one susceptible variant alongside additional variants with unknown sensitivity. Together, our findings suggest that 34 HLA-A∗02:01-positive individuals harbouring Gag^77–85^ sequences predicted to be recognised by IMC-M113V may represent potential candidates for this therapy, corresponding to 41.0% of all participants.

## Discussion

To advance both treatment perspective and curative efforts, a detailed understanding of the persisting HIV-1 reservoir composition is crucial. This need is becoming increasingly pressing, as recent reports warn that AIDS-related deaths may rise due to disruptions in ART supply chains, conflicts, declining ART-adherence and accelerating risk of novel DRMs.^67^^,^^68^ In this context, comprehensive reservoir characterisation will be essential to monitor viral evolution, guide therapeutic strategies and assist progress toward durable HIV remission. Here, we applied the recently developed and cost-effective HIV-PULSE to extensively profile the latent HIV-1 reservoir in 83 individuals from the 2000HIV cohort. By integrating diverse analytical approaches, we gain valuable insights into the composition of the viral reservoir and potential immune therapy targets.

This is one of the first studies determining the proviral landscape in a large cohort by classifying 4040 unique proviral sequences as predicted intact or defective. Consistent with prior studies, the majority of proviruses were defective, with ∼4% classified as putatively intact.^69^, ^70^, ^71^ Of these intact proviruses, 15.3% were detected in multiple replicates, suggesting clonality and highlighting the persistence of the intact viral reservoir on ART.^72^^,^^73^ While HIV-PULSE does not capture integration site data, the use of replicate-based detection provides supportive evidence for clonality. However, accurate quantification of clonality is lacking as the estimate is likely an underrepresentation of the true clonal size. Additionally, we observed intra-individual phylogenetic diversity among intact genomes. These findings underscore that relying on a single intact genome or consensus sequence underestimate the true reservoir heterogeneity, whereas capturing multiple proviruses offers more comprehensive insights.

Comparison of HIV-PULSE with conventional genotyping approaches for tropism prediction and subtyping further extends these observations. Traditional population-based genotyping typically targets a limited genomic region, such as *env*, and relies on Sanger sequencing.^74^^,^^75^ In contrast, our data demonstrate that NFL sequencing across multiple proviruses captures genetic variation that remains unresolved by partial *env*-based genotyping. Relative to *env* Sanger sequencing, HIV-PULSE detected minority variants within mixed-tropic proviral populations and enabled refined subtype determination based on NFL genomic context. The enhanced sensitivity of HIV-PULSE to identify minority X4-tropic proviruses is particularly relevant when evaluating individuals for R5-directed therapeutic interventions, as the presence of X4 variants may undermine treatment efficacy.

Beyond its applications in genotyping and functional reservoir profiling, HIV-PULSE can also inform the design and optimisation of reservoir quantification assays.^43^ By mapping the primers and probes used in dPCR-based assays, like the Rainbow HIV-1 DNA assay, against the NFL proviral sequences from each individual, HIV-PULSE enables the evaluation of primer-template compatibility and the identification of potential mismatches that could impair detection. In cases where binding sites are affected, the data can guide the development of individualised primer or probe sets, thereby enhancing assay sensitivity and accuracy. This approach not only refines quantitative reservoir measurements but also illustrates how NFL-informed assay customisation could help primer design for other sequencing assays and support more precise and personalised intervention strategies.

Leveraging the methodological strengths of HIV-PULSE, we next characterised the extent and determinants of proviral diversity revealed through NFL sequencing. Evaluating this diversity is clinically important, as certain treatments must neutralise a range of proviral variants. Nevertheless, three participants exhibited higher diversity (>4%), including one individual with phylogenetic evidence suggesting multiple infections. Clustering of three distinct proviruses was consistently observed across multiple genomic regions. This clustering pattern was less distinct in the *env* region, possibly reflecting recombination. As longitudinal samples were not available, a distinction between dual and superinfection cannot be made. While superinfections are rare, cases have been documented even in chronic infections.^76^, ^77^, ^78^, ^79^ Notably, all three individuals also experienced low-level viraemia or viral blips in the three years prior to sampling, raising the possibility that intermittent viral replication contributed to within-host diversity during ART. Furthermore, multivariate modelling confirmed the association between genetic diversity and delayed ART initiation, lower pre-treatment CD4 counts and the presence of mixed R5/X4 tropism. The coexistence of R5 and X4 variants within an individual reflects the genetic variation in the HIV-1 envelope region and the heterogeneous viral population. Higher baseline CD4 counts often indicate shorter time between infection and ART initiation, correlating with a less diverse reservoir.^80^ These findings support the consensus that reservoir diversity during ART is predominantly shaped by pre-ART factors such as time since infection and pre-treatment viral replication.^80^, ^81^, ^82^, ^83^, ^84^

Current clinical HIV-1 drug resistance testing relies on plasma RNA sequencing, which reflects the actively replicating viral populations.^15^^,^^85^ However, proviral DNA sequencing has increasingly emerged as a tool for assessing resistance, particularly in settings with suppressed viraemia or screenings for archived resistance.^15^^,^^86^, ^87^, ^88^ Here, we screened for DRMs using HIV-PULSE derived sequences. Despite excluding hypermutated sequences from this analysis, careful interpretation remains necessary for HIV-1 proviral resistance genotyping.^15^ DRMs were identified in 16.5% of sequences, spanning 53 participants. While these may overestimate the prevalence of circulating variants, they highlight the presence of archived resistance. Discrepancies between HIV-PULSE sequences and pre-ART plasma genotyping, such as the detection of DRMs in proviral genomes that were absent pre-ART, may reflect selective pressure during therapy, emergence of minority variants below the detection threshold of Sanger-based screenings, or naturally occurring polymorphisms. By focussing exclusively on intact proviruses, we wanted to achieve the best possible estimate of potential replication-competent proviruses. Among seven participants with a pre-ART DRM, two harboured intact proviruses containing the same resistance mutations after years of ART. This supports the notion that archived drug-resistant variants can persist over time despite ART. Notably, while Sanger-based plasma RNA sequencing remains the gold standard in clinical practice, it primarily captures the most abundant plasma viral variants and cannot resolve whether multiple resistance mutations co-localise on the same genome. By contrast, HIV-PULSE provides single-genome resolution, enabling improved detection of minority and combinatorial resistance profiles. These insights illustrate how long-read sequencing of proviruses can reveal a broad picture of the resistance profile in the viral reservoir, thereby providing complementary insights with potential clinical implications, such as informing treatment modifications in virally suppressed individuals.

To evaluate the potential of immune-based therapies to target the reservoir, we performed in silico sensitivity predictions to bNAbs and the Gag-targeting ImmTav IMC-M113V. Although applied in clinical trials, the PhenoSense Monoclonal Antibody Assay is expensive, labour-intensive, and exhibits inconsistent predictive capacity.^89^ Genotypic approaches represent a viable alternative, typically relying on sequencing partial or complete *env* regions from plasma RNA.^90^, ^91^, ^92^, ^93^, ^94^ Multiple clinical trials are evaluating bNAbs for both treatment and prevention of HIV-1 infection, with combinations of 3BNC117 and 10–1074, VRC07-variants, or N6LS.^64^, ^65^, ^66^^,^^95^ In our cohort, a subset of participants harboured proviral genomes (both intact and defective ones) predicted to be sensitive to some of these bNAbs, suggesting that they could potentially meet inclusion criteria for ongoing or future bNAb-based clinical studies. This highlights how genotyping with HIV-PULSE can extend beyond reservoir characterisation to inform clinical trial stratification and selection of candidates most likely to benefit from antibody-based interventions. Beyond standard *env*-based genotyping, our NFL approach further links bNAb sensitivity screening to proviral genomic integrity. Our results indicate that defective proviruses are predicted to be more likely resistant to bNAbs. However, PSI/MSD defective proviruses demonstrated neutralisation profiles closely resembling those of intact proviruses. This observation aligns with their 5′-localised defects, which retains *env* conformations necessary for bNAb recognition. As PSI/MSD defective proviruses can contribute to chronic immune activation,^96^^,^^97^ clearing cells harbouring these proviruses may offer clinical benefit by reducing inflammation. Our data highlight that proviral diversity contributes to the heterogeneity of neutralising effects observed within participants. Although previous studies demonstrated that both phenotypic and genotypic sensitivity analyses have limited capacity to predict clinical responses to bNAbs,^64^^,^^98^ a genotypic strategy like HIV-PULSE may nevertheless inform the design of bNAb-based interventions by providing *env* sequence information in the context of proviral intactness.

Similarly, for immune-based interventions targeting *gag*, sensitivity to IMC-M113V was predicted by analysing the presence of Gag^77–85^ epitope variants.^32^ Although the Gag^77–85^ region is generally more conserved than *env* and in vitro and preclinical studies have demonstrated the potency of IMC-M113V against eight Gag^77–85^ epitope variants,^31^^,^^32^ our data revealed considerable variability in epitope composition both within and between individuals. As the IMC-M113V therapy relies on HLA-restricted antigen presentation, only individuals who are HLA-A∗02:01 positive and harbour sensitive Gag^77–85^ variants would be eligible for this therapy. Altogether, our findings indicate that a consensus-based approach may underestimate the heterogeneity of the viral reservoir, underscoring the need for individualised sequence data to inform immune therapeutic strategies.

Our study has several limitations that reflect the challenges in interpreting proviral DNA data. Although HIV-PULSE enables high-throughput recovery of NFL proviruses and provides valuable insights into intactness and therapeutic susceptibility, the method cannot determine whether these proviruses are truly replication-competent, which ultimately requires functional assays. In addition, the sensitivity for detecting intact proviruses is limited, with intact proviruses identified in 59% of all participants. This likely reflects sampling bias inherent to NFL sequencing approaches, which capture only a fraction of the total reservoir.^99^ As a result, rare proviruses or specific genetic features may be missed. For example, the absence of multiple DRMs within the same proviral sequence does not exclude the possibility that such combinations exist elsewhere in the reservoir. Therefore, the findings should be interpreted within the context of this sampling limitation. Furthermore, limited sample availability restricted the number of participants from the 2000HIV cohort included in this study and prevented re-analysis of samples with suboptimal PCR yield. Finally, our predictions for coreceptor usage and sensitivity to bNAbs and ImmTavs are based solely on sequencing characteristics. These predictions may not fully capture the complexity of in vivo activity, including the influence of the local immune environment and structural-functional features that cannot be accurately inferred from sequence data alone. Previous studies have shown that genotypic bNAb sensitivity analyses have limited capacity to predict clinical responses to bNAbs,^64^^,^^98^ underscoring the need for improved, reliable computational tools. Nonetheless, HIV-PULSE demonstrated high overall success rate, with proviral sequences obtained from 83 participants, representing a sizeable cohort for single proviral reservoir studies, even when starting from a relatively small cell input (495,000 CD4 cells per individual) and including those in lower-range viral reservoir sizes (134–300 copies per million CD4 cells).

In conclusion, our findings provide a multidimensional view of the genetic and functional landscape of HIV-1 persistence, derived from sequences generated through a single assay. By combining structural classification with screening predictions for drug resistance and immune-based therapies, we highlight the added value of HIV-PULSE as a framework for characterising HIV-1 reservoir composition and informing cure and treatment strategies. Such improved predictive capacity not only supports the design of more effective studies but also contributes to safer patient outcomes and more efficient use of research resources.

## Contributors

S.D., L.L., S.R., S.G., and L.V. conceptualised the experiments. S.D. and L.T. performed experiments. M.D. performed HIV-1 DNA measurements. K.M. performed HIV-1 env Sanger sequencing. S.D. and L.L. analysed viral sequencing data. V.M. performed HLA-typing. P.L. and B.V. gave additional scientific input. E.B., C.R., J.S., M.B., and A.J.V. coordinated the study. S.D., S.R., and L.V. had access to the underlying data. S.D. made figures and tables. S.D. wrote the manuscript. All co-authors edited and approved the manuscript.

## Data sharing statement

HIV-1 sequencing data supporting the findings of this study have been deposited in GenBank with the accession code: PX947546-PX951409. The scripts used in this study are publicly available on GitHub: https://github.com/HCRCugent/longread_umi_hiv. The GitHub page provides a description of the key operations and instructions on how to install and run the code. The data have also been deposited in the Radboud Data Repository (https://doi.org/10.34973/3292-1m86). Individual clinical data from the 2000HIV cohort used in this manuscript will be made publicly available following the conclusion of the 2000HIV study in 2026. Request for data access can be directed to the committee via datarequests.aig@radboudumc.nl.

## Declaration of interests

S.D., M.D., K.M., and E.B received a “New Investigators Scholarship” to attend the *Conference on Retroviruses and Opportunistic Infections*. S.D., L.L., P.L. received grants by FWO Vlaanderen. C.R. and L.V. received support from Gilead, ViiV Healthcare, and MSD. C.R. has served in the NEAT advisory board and holds stocks in Immunocore. The remaining authors declare that they have no competing interests.

## Appendix A Supplementary data

Supplementary Tables and Figures

SupplementaryTable 6
