# Supplementary material for: HIV-1 virome profiling using HIV-PULSE to guide therapeutic and curative interventions
Source: eBioMedicine. 2026 May 21;128:106297. doi: 10.1016/j.ebiom.2026.106297 (PMC13199670; doi:10.1016/j.ebiom.2026.106297)
Supplement: Supplementary Tables and Figures [file mmc1.docx]

# Supplementary Figures


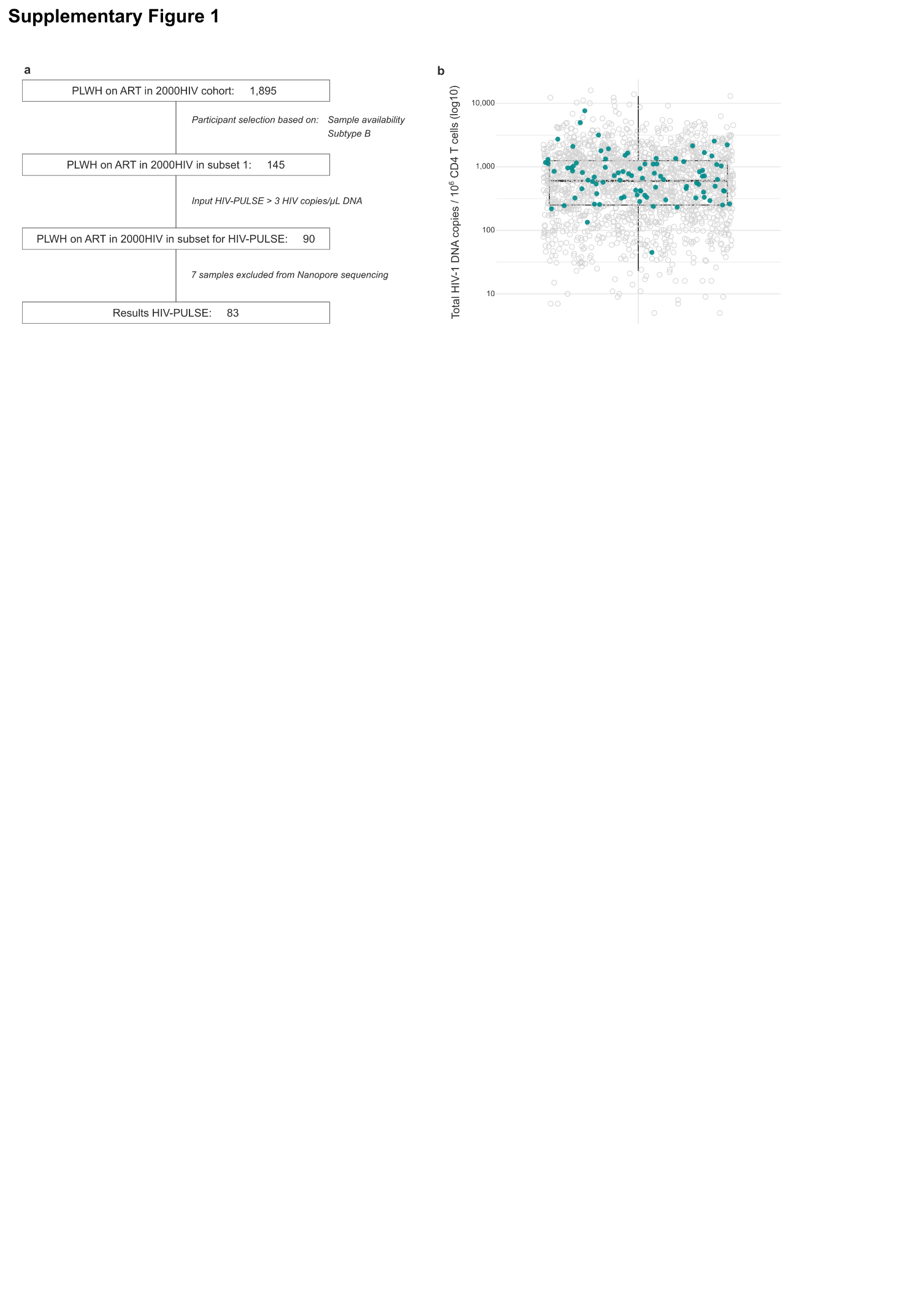


**Supplementary Figure 1. Workflow HIV-PULSE in 2000HIV cohort**HIV-PULSE sequencing was performed on peripheral blood samples from 83 participants in the 2000HIV cohort. a) Participants were selected based on sample availability, subtype B and an HIV-PULSE input exceeding 3 copies/µL DNA. Seven samples were excluded from Nanopore sequencing due to insufficient DNA concentration for library preparation. b) Total HIV-1 reservoir size across the 2000HIV cohort, measured as HIV-1 DNA copies per 10^6^ CD4 T cells. Blue dots represent the 83 participants with HIV-PULSE sequences included in this study.


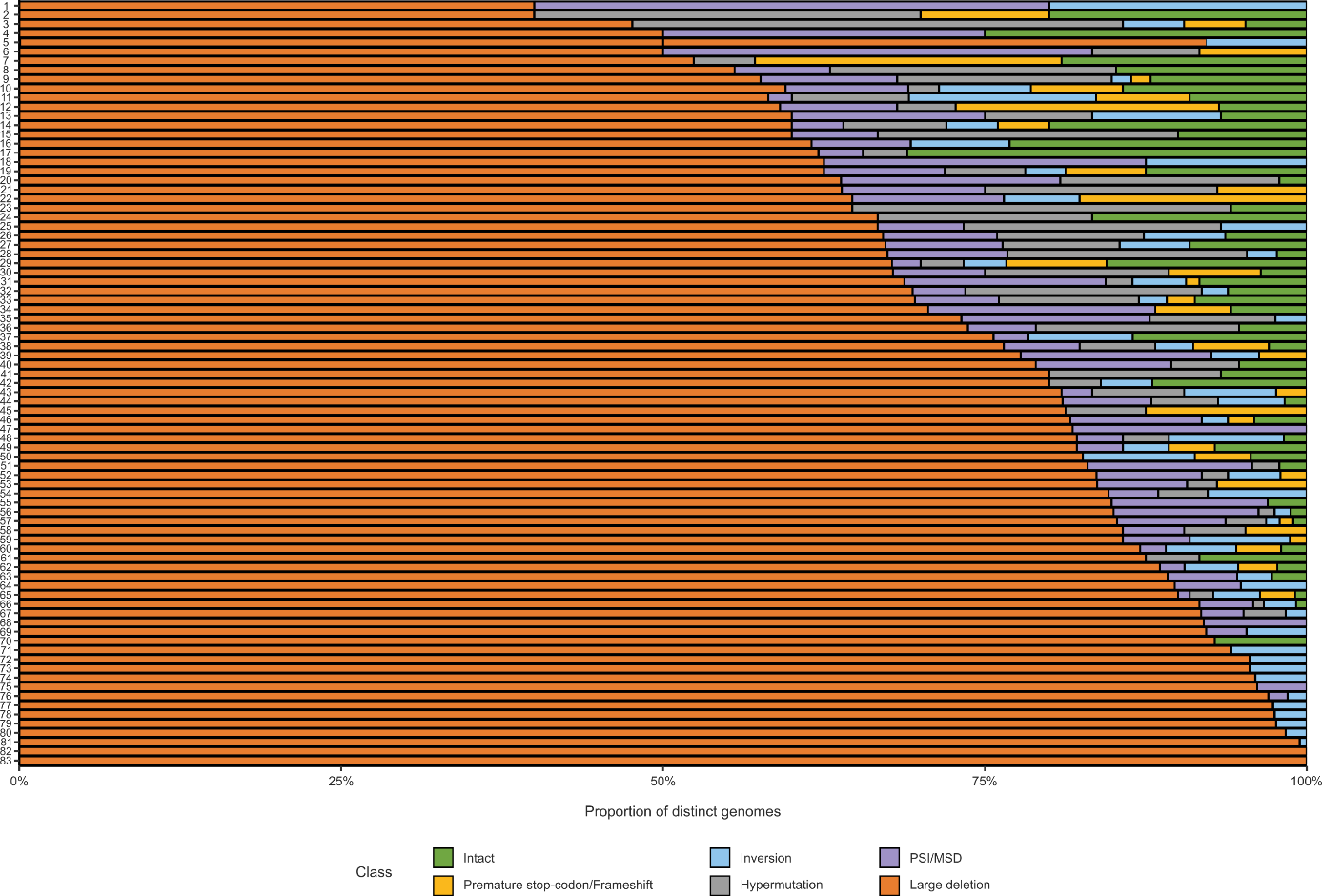


**Supplementary Figure 2. Proviral reservoir characterization in all participants**The proviral reservoir assayed by the HIV-PULSE assay in 83 participants. The barplot shows the proportions of different proviral classes observed among the distinct proviral genomes for each participant.


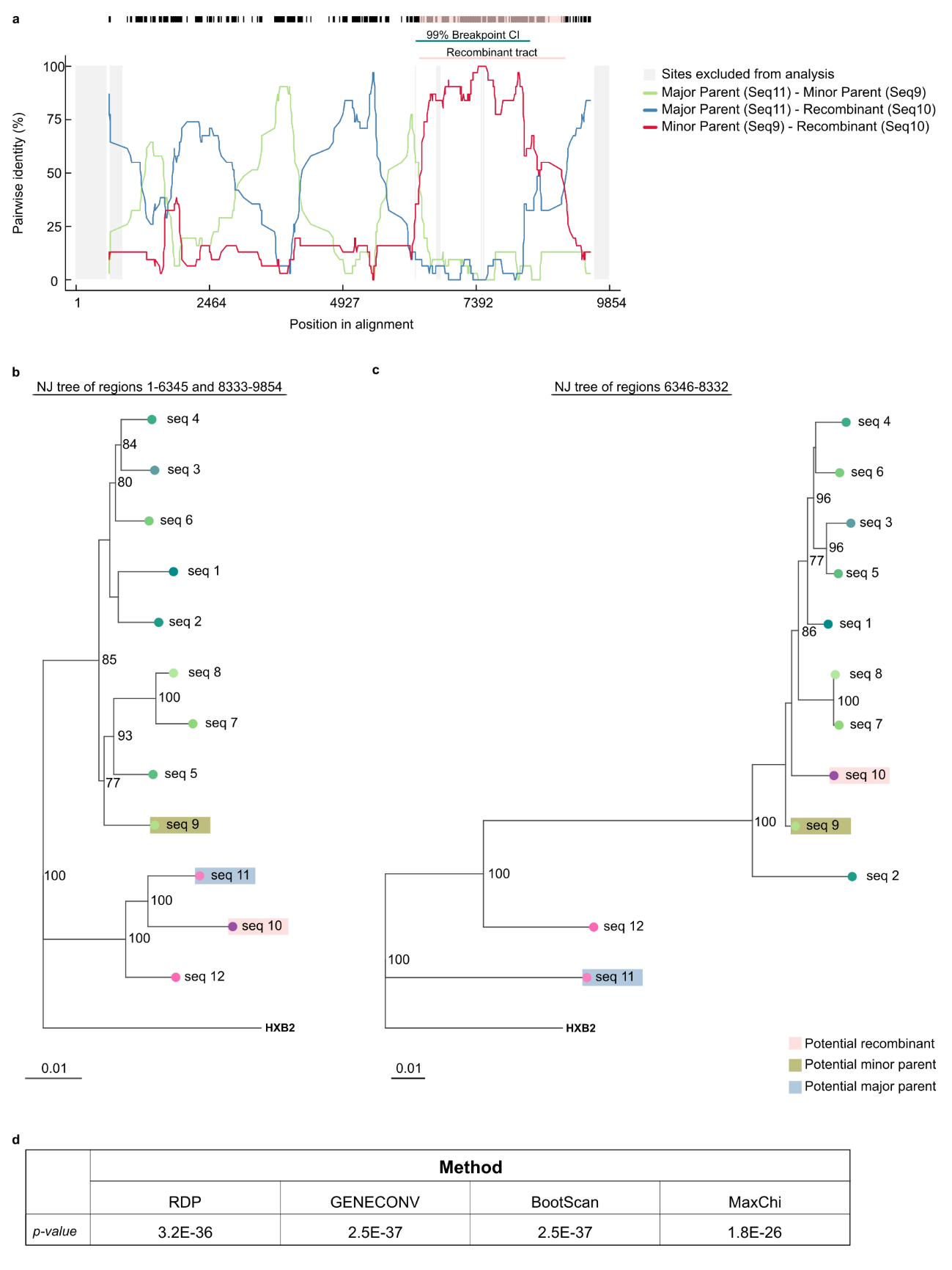


**Supplementary Figure 3. Recombination analysis**
Recombination between two viral strains within one individual was assessed using the RDP5 software. a) Schematic overview of the recombination event predicted by RDP5 across the proviral genome. b-c) Neighbour-joining phylogenetic trees illustrating the potential recombinant sequence and putative parental lineages for genomic regions 1-6,345 and 8,333-9,854 (b), and 6,346-8,332 (c). d) Statistical support for the detected recombination events based on multiple methods implemented in RDP5.

**
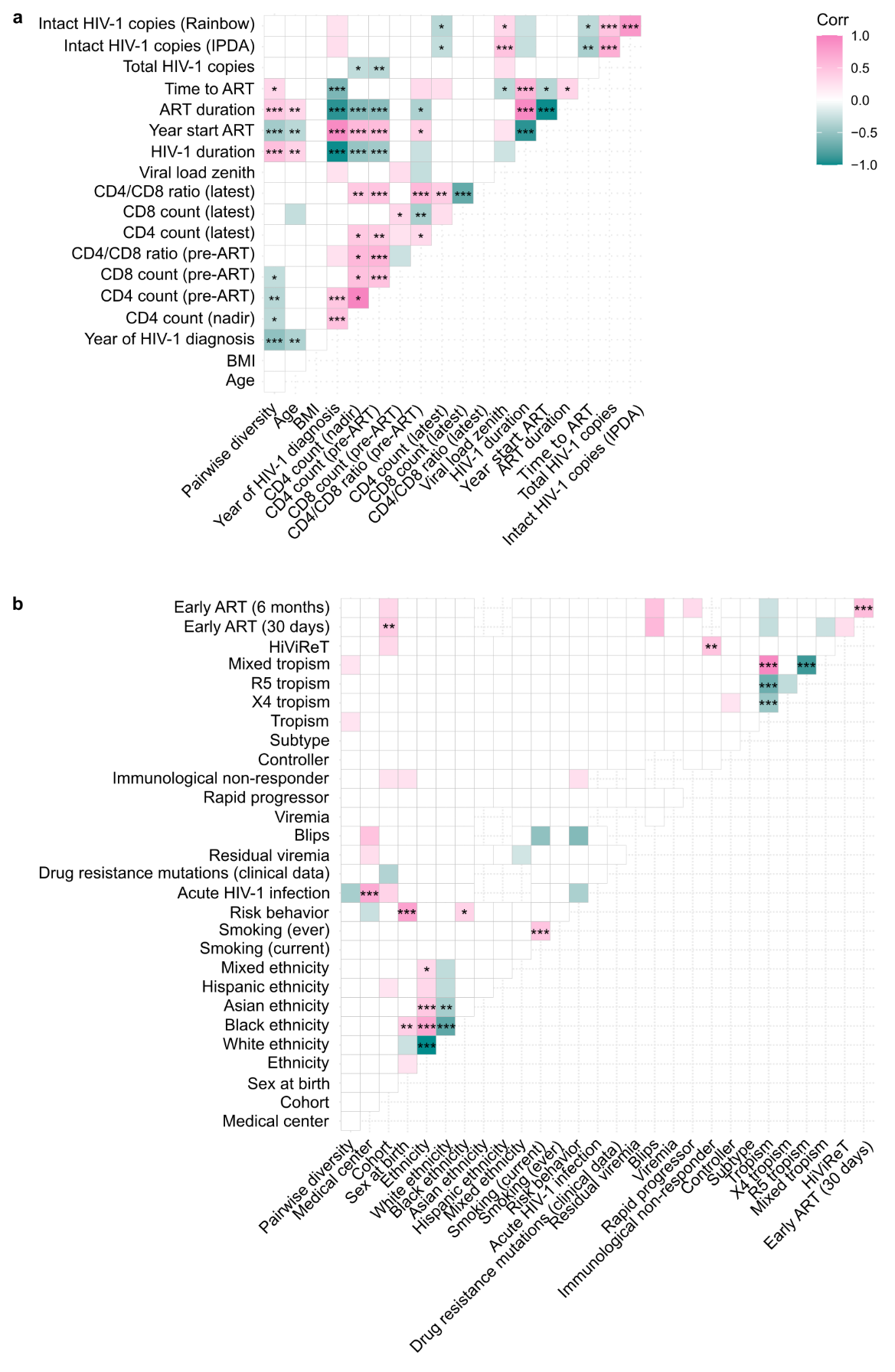
**

**Supplementary Figure 4. Clinical correlates of proviral reservoir diversity**
Correlogram of univariate spearman correlations between clinical characteristics (continuous (a) and categorical (b)). Colored squares indicate significant correlations, while asterisks denote significance after multiple-testing correction (Benjamini-Hochberg): * p_adj_ < 0·05; ** p_adj_ < 0·01; *** p_adj_ < 0·001. Terms are explained in the glossary (Supplementary Table 2).

**
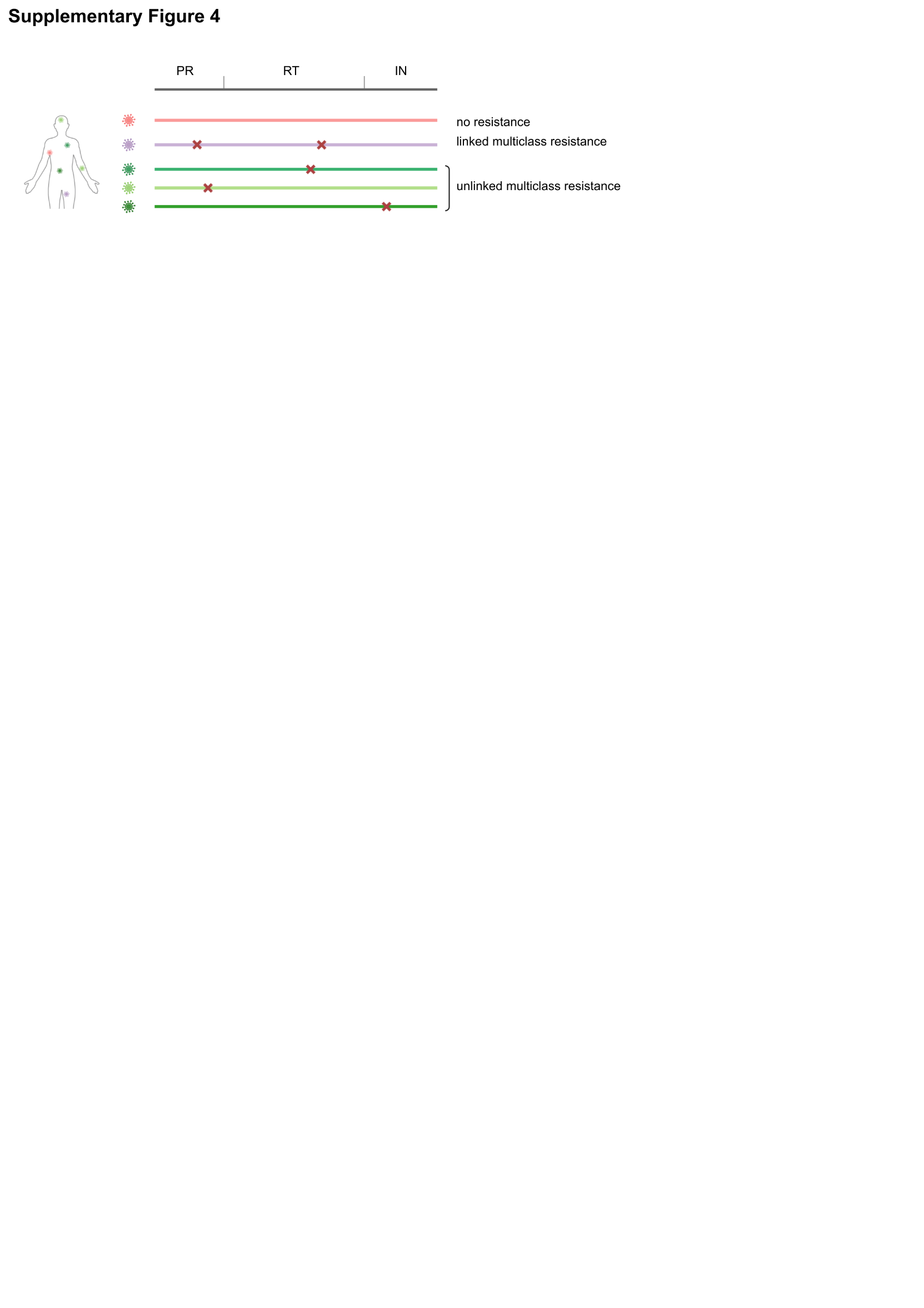
**

**Supplementary Figure 5. Visual representation of multiclass drug resistance profiles**Schematic overview illustrating the different patterns of drug resistance mutations predicted across multiple antiretroviral drug classes.

**
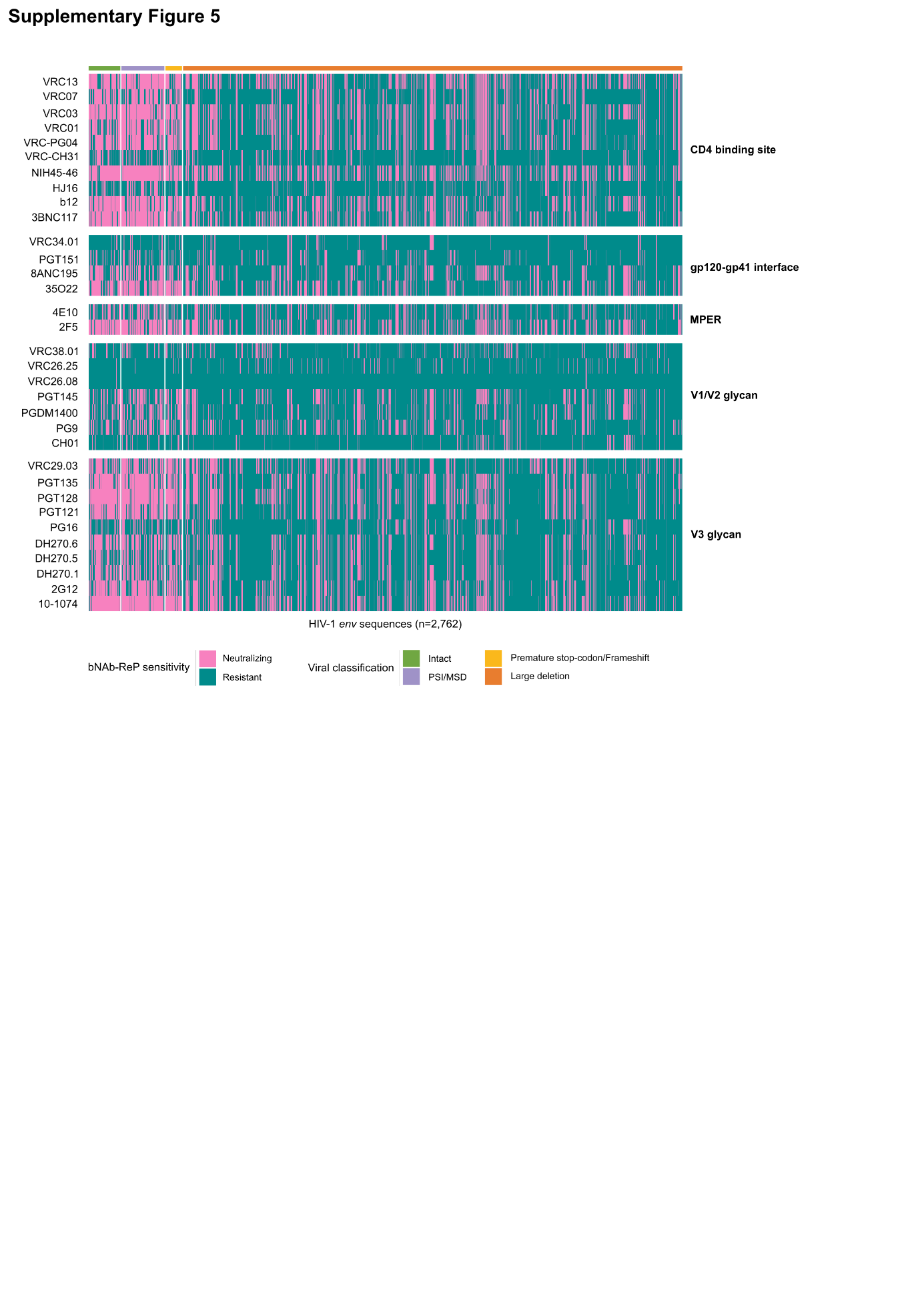
**

**Supplementary Figure 6. In silico bNAb sensitivity prediction for all proviral sequences**Predicted bNAb resistance based on bNAb-ReP predictions from HIV-1 *env* proviral sequences from 2,762 proviruses, including intact and defective proviruses (PSI/MSD defects, large deletions and premature stop codon or frameshift). Results are grouped by bNAb binding site. Terms are explained in the glossary (Supplementary Table 2). MPER, membrane-proximal external region.

# Supplementary Tables

**Supplementary Table 1. Additional clinical characteristics of the study participants**Terms are explained in the glossary (Supplementary Table 2).

| **Characteristic** | **2000HIV** *N=90* |
| --- | --- |
|  | n (%) |
| Smoking current |  |
| Non-smoker | 52 (57·8%) |
| Smoker | 30 (33·3%) |
| Unknown | 8 (8·9%) |
| Smoking ever |  |
| No | 23 (25·6%) |
| Yes | 59 (65·6%) |
| Unknown | 8 (8·9%) |
| Acute HIV |  |
| No | 24 (26·7%) |
| Yes | 23 (25·6%) |
| Unknown | 43 (47·8%) |
| Early ART (6 months) |  |
| No | 39 (43·8%) |
| Yes | 32 (35·6%) |
| Unknown | 19 (21·1%) |
| Early ART (30 days) |  |
| No | 57 (63·3%) |
| Yes | 14 (15·6%) |
| Unknown | 19 (21·1%) |
| Viremia |  |
| No | 20 (22·2%) |
| Yes | 1 (1·1%) |
| Unknown | 69 (76·7%) |
| Blips |  |
| No | 8 (8·9%) |
| Yes | 13 (14·4%) |
| Unknown | 69 (76·7%) |
| Residual viremia |  |
| No | 21 (23·3%) |
| Yes | 69 (76·7%) |
| HiViReT |  |
| No | 84 (93·3%) |
| Yes | 6 (6·7%) |
| Controller |  |
| No | 86 (95·6%) |
| Yes | 3 (3·3%) |
| Unknown | 1 (1·1%) |
| Immunological non-responder |  |
| No | 81 (90·0%) |
| Yes | 1 (1·1%) |
| Unknown | 8 (8·9%) |
| Rapid progressor |  |
| No | 50 (55·6%) |
| Yes | 5 (5·56%) |
| Unknown | 35 (38·9%) |
| Risk behavior |  |
| IV drug use | 1 (1·1%) |
| Heterosexual | 21 (23·3%) |
| MSM | 64 (71·1%) |
| Unknown | 4 (4·4%) |

**Supplementary Table 2. Glossary of terms**

| **Terms** | **Explanation** |
| --- | --- |
| Acute HIV | A recent HIV-1 infection at diagnosis. |
| ART duration (years) | The number of years since a participant has been on ART. |
| Blips | Temporary, detectable increase in HIV-1 viral load, between quantification lower limit and 200 copies/mL, during the 3 years prior to baseline. |
| CD4 latest | The most recent CD4 T cell count at baseline. |
| CD4 nadir | The lowest CD4 T cell count ever. |
| CD4 pre-ART | The most recent CD4 T cell count before start ART. |
| CD4/CD8 ratio latest | The most recent CD4/CD8 ratio at baseline. |
| CD4/CD8 ratio pre-ART | The most recent CD4/CD8 ratio before start ART. |
| CD8 latest | The most recent CD8 T cell count at baseline. |
| CD8 pre-ART | The most recent CD8 T cell count before start ART. |
| Center (/ Medical center) | Participants in the 2000HIV study were recruited from four specialized Dutch HIV treatment centers: Radboudumc Nijmegen, Erasmus MC Rotterdam, OLVG Amsterdam and Elisabeth-TweeSteden Ziekenhuis Tilburg. |
| Cohort | The 2000HIV study consists of a discovery and validation cohort. |
| Controller | Person living with HIV-1 who is able to maintain undetectable viral load currently or in the past, with and without ART. |
| Drug resistance mutations (based on clinical file) | HIV-1 mutations conferring ART-resistance before start ART, based on the clinical file. |
| Drug resistance mutations (DRM) | HIV-1 mutations conferring potential low- to high-level resistance, based on HIV-PULSE sequences. |
| Early ART (30 days) | After confirmed acute HIV-1 infection, the participant initiated ART within 1 month. |
| Early ART (6 months) | After confirmed acute HIV-1 infection, the participant initiated ART within 6 months. |
| HIV-1 duration | The number of years since a participant was diagnosed with HIV-1. |
| HiViReT | People living with HIV-1 who are having the highest 10% total HIV-1 reservoir size, exceeding 2,200 copies/10^6^ CD4 cells. |
| Immune-mobilizing monoclonal T cell receptors against virus (ImmTAV) | Bispecific molecules designed to enable the immune system to recognize and eliminate virally infected cells. |
| Immunological non-responder | Participant on ART for at least 2 years that has a CD4 T cell count below 350. |
| INSTI class | Individual currently using integrase strand inhibitors. |
| Intact HIV-1 DNA copy number - IPDA | The intact HIV-1 DNA reservoir size, based on the *psi* and *env* region. |
| Intact HIV-1 DNA copy number - Rainbow | The intact HIV-1 DNA reservoir size, based on the *psi*, *pol*, *gag* and *env* region. |
| Mixed tropism | Proviral reservoir composed of a mixture of R5-tropic and X4-tropic viruses. |
| Membrane-Proximal External Region (MPER) | Specific region on the HIV-1 gp41 fusion protein targeted by broadly neutralizing antibodies. |
| NNRTI class | Individual currently using non-nucleoside reverse transcriptase inhibitors. |
| NRTI class | Individual currently using nucleoside reverse transcriptase inhibitors. |
| PI class | Individual currently using protease inhibitors. |
| Polymorphism | Resistance mutation not associated with reduced susceptibility to antiretrovirals. |
| Rapid progressor | Person living with HIV-1 that rapidly progresses to AIDS if they fail to take ART within 4 years after primary HIV-1 infection. |
| Residual viremia | A quantifiable viral load of > 40 copies/mL during the 3 years prior to baseline. |
| Risk behavior | Risk behavior associated with HIV transmission. |
| Smoking current | Current smoking status of the participant. |
| Smoking ever | Smoking status of participant ever, currently or in the past. |
| Time to ART | The number of days between HIV-1 diagnosis and start of ART. |
| Total HIV-1 DNA copy number | The total HIV-1 reservoir size, measured by a qPCR of the repeated unique 5' target region. |
| Viral load zenith | The highest viral load measurement ever. |
| Viremia | Increase in viral load between 200 and 1,000 copies/mL during the 3 years prior to baseline. |

**Supplementary Table 3. Representativeness of the subgroups**Comparison of clinical and virological characteristics of study subgroups with the overall 2000HIV cohort. Continuous variables were compared using the Wilcoxon rank-sum test and categorical variables using the Chi-square or Fisher’s exact test. P-values were adjusted using the Benjamini-Hochberg method: * p_adj_ < 0·05; ** p_adj_ < 0·01; *** p_adj_ < 0·001.

|  | **2000HIV cohort** | **Subset 1** | | **Subset 2** | | **Subset 3** | |
| --- | --- | --- | --- | --- | --- | --- | --- |
| **Characteristic** |  | HIV-PULSE selection | | HIV-PULSE sequenced | | Pre-ART plasma samples | |
|  | *N=1879* | *N=90* | *p_adj_* | *N=83* | *p_adj_* | *N=20* | *p_adj_* |
| Age in years, median (IQR) | 53 (44-60) | 54 (45-58) |  | 54 (45·5-58) |  | 50 (45·8-56·5) |  |
| BMI at baseline (kg/m2), median (IQR) | 25·0 (22·4-27·7) | 24·8 (22·7-27·1) |  | 24·8 (22·9-27·2) |  | 25·5 (24·6-26·7) |  |
| HIV duration in years, median (IQR) | 12·4 (7·1-19·1) | 13·7 (9·3-20·0) |  | 13·7 (9·7-20·6) |  | 11·6 (9·7-13·4) |  |
| ART duration in years, median (IQR) | 10·1 (5·9-16·3) | 11·7 (7·3-15·4) |  | 11·8 (7·4-15·3) |  | 9·7 (7·2-11·7) |  |
| Viral load zenith in copies/ml, median (IQR) | 100,000 (38,950 - 285,762) | 125,000 (70,120-287,588) |  | 119,000 (68,083-218,805) |  | 155,357 (47,840-171,500) |  |
| CD4 nadir (10^9^/mL), median (IQR) | 0·3 (0·1-0·4) | 0·3 (0·2-0·4) |  | 0·3 (0·2-0·4) |  | 0·3 (0·3-0·4) |  |
| CD4 latest (10^9^ cells/mL), median (IQR) | 0·7 (0·5-0·9) | 0·9 (0·6-1·1) | ** | 0·9 (0·6-1·1) | ** | 0·9 (0·8-1·1) | * |
| CD8 latest (10^9^ cells/mL), median (IQR) | 0·8 (0·6-1·2) | 0·8 (0·6-1·2) |  | 0·8 (0·6-1·2) |  | 0·7 (0·6-1·0) |  |
| Total HIV-1 DNA copy number (copies/Mio CD4 cells), median (IQR) | 600 (250·3-1,244) | 712 (404-1,111) |  | 727·5 (422-1120) |  | 747·5 (330-991·8) |  |
| Intact HIV-1 DNA copy number (copies/Mio CD4 cells), median (IQR) | 16 (4-51) | 32 (7-58·5) |  | 29 (7-58·8) |  | 11 (3·5-51·5) |  |
| Sex at birth, N (%) |  |  |  |  |  |  | *** |
| *Male* | 1595 (84·9%) | 76 (84·4%) |  | 70 (84·3%) |  | 19 (95·0%) |  |
| *Female* | 284 (15·1%) | 14 (15·6%) |  | 13 (15·7%) |  | 1 (5·0%) |  |
| Ethnical ancestry, N (%) |  |  |  |  |  |  | ** |
| *White* | 1419 (75·5%) | 79 (87·8%) |  | 73 (88·0%) |  | 20 (100%) |  |
| *Black* | 189 (10·1%) | 7 (7·8%) |  | 6 (7·2%) |  | 0 (0%) |  |
| *Asian* | 86 (4·6%) | 2 (2·2%) |  | 2 (2·4%) |  | 0 (0%) |  |
| *Hispanic* | 49 (2·6%) | 1 (1·1%) |  | 1 (1·2%) |  | 0 (0%) |  |
| *Native American* | 3 (0·2%) | 0 (0%) |  | 0 (0%) |  | 0 (0%) |  |
| *Mixed* | 131 (7·0%) | 1 (1·1%) |  | 1 (1·2%) |  | 0 (0%) |  |
| *Unknown* | 2 (0·1%) | 0 (0%) |  | 0 (0%) |  | 0 (0%) |  |
| Smoking status, N (%) |  |  |  |  |  |  |  |
| *Current smoker* | 554 (29·5%) | 30 (33·3%) |  | 26 (31·3%) |  | 6 (30·0%) |  |
| *Previous smoker* | 567 (30·2%) | 29 (32·2%) |  | 27 (32·5%) |  | 4 (20·0%) |  |
| *Never smoked* | 617 (32·8%) | 23 (25·6%) |  | 22 (26·5%) |  | 9 (45·0%) |  |
| *Unknown* | 141 (7·5%) | 8 (8·9%) |  | 8 (9·6%) |  | 1 (5·0%) |  |
| Mode of transmission, N (%) |  |  |  |  |  |  | *** |
| *MSM* | 1350 (71·8%) | 64 (71·1%) |  | 60 (72·3%) |  | 16 (80·0%) |  |
| *Heterosexual contact* | 405 (21·6%) | 21 (23·3%) |  | 20 (24·1%) |  | 3 (15·0%) |  |
| *Intravenous drug use* | 20 (1·1%) | 1 (1·1%) |  | 1 (1·2%) |  | 0 (0%) |  |
| *Congenital* | 11 (0·6%) | 0 (0%) |  | 0 (0%) |  | 0 (0%) |  |
| *Contaminated blood products* | 6 (0·3%) | 0 (0%) |  | 0 (0%) |  | 0 (0%) |  |
| *Unknown* | 87 (4·6%) | 4 (4·4%) |  | 2 (2·4%) |  | 1 (5·0%) |  |
| ART classes in use, N (%) |  |  |  |  |  |  |  |
| *NRTI* | 1795 (95·5%) | 90 (100%) |  | 83 (100%) |  | 20 (100%) | *** |
| *NNRTI* | 737 (39·2%) | 45 (50·0%) |  | 41 (49·4%) |  | 9 (45·0%) |  |
| *PI* | 179 (9·5%) | 4 (4·4%) |  | 3 (3·6%) |  | 0 (0%) |  |
| *INSTI* | 1023 (54·4%) | 42 (46·7%) |  | 40 (48·2) |  | 11 (55·0%) |  |
| Extreme HIV phenotypes, N (%) |  |  |  |  |  |  |  |
| *Spontaneous controller* | 114 (6·1%) | 3 (3·3%) |  | 3 (3·6%) |  | 1 (5·0%) |  |
| *Immunological non-responder* | 88 (4·7%) | 1 (1·1%) |  | 1 (1·2%) |  | 0 (0%) |  |
| *Rapid progressor* | 80 (4·3%) | 5 (5·56%) |  | 4 (4·8%) |  | 2 (10·0%) |  |
| Diagnosis during acute HIV infection, N (%) | 417 (22·2%) | 23 (25·6%) |  | 19 (22·9%) |  | 8 (40·0%) |  |
| Early ART (i·e· ART within first month of diagnosis of recent HIV infection), N (%) | 87 (4·6%) | 14 (15·6%) |  | 14 (16·9%) |  | 2 (10·0%) |  |

**Supplementary Table 4. List of primers used in this study**

| **Assay** | **Reference** | **Amplicon** | **HXB2 coordinates** | **Forward/Reverse** | **Round** | **Primer** | **Sequence (5' to 3')** |
| --- | --- | --- | --- | --- | --- | --- | --- |
| HIV-PULSE | Lambrechts, et al. NAR. 2023 | Pre-amplification | 523-9591 | Forward | 1 | First PCR F | CCTCAATAAAGCTTGCCTTGAGTGC |
|  |  |  |  | Reverse | 1 | First PCR R | CCTAGTTAGCCAGAGAGCTCCCAG |
|  |  | Tagging | 551-9458 | Forward | 1 | Second PCR F UMI | CAAGCAGAAGACGGCATACGAGATNNNYRNNNYRNNNYRNNNAAGTAGTGTGTGCCCGTCTGTTGTGTGAC |
|  |  |  |  | Reverse | 1 | Second PCR R UMI | AATGATACGGCGACCACCGAGATCNNNYRNNNYRNNNYRNNNGGAAAGTCCCCAGCGGAAAGTCCCTTGTAG |
|  |  | Amplification | NA | Forward | 1-2-3 | ncec_pcr_fw_v7 | CAAGCAGAAGACGGCATACGAGAT |
|  |  |  |  | Reverse | 1-2-3 | ncec_pcr_rv_v7 | AATGATACGGCGACCACCGAGATC |
|  |  | Amplification ID1 | NA | Forward | 4 | ONT_ID1_fw1 | ACGAGACTGATTCAAGCAGAAGACGGCATACGAGAT |
|  |  |  |  | Reverse | 4 | ONT_ID1_rv1 | ACGAGACTGATTAATGATACGGCGACCACCGAGATC |
|  |  | Amplification ID2 | NA | Forward | 4 | ONT_ID2_fw2 | GCTGTACGGATTCAAGCAGAAGACGGCATACGAGAT |
|  |  |  |  | Reverse | 4 | ONT_ID2_rv2 | GCTGTACGGATTAATGATACGGCGACCACCGAGATC |
|  |  | Amplification ID3 | NA | Forward | 4 | ONT_ID3_fw3 | ATCACCAGGTGTCAAGCAGAAGACGGCATACGAGAT |
|  |  |  |  | Reverse | 4 | ONT_ID3_rv3 | ATCACCAGGTGTAATGATACGGCGACCACCGAGATC |
|  |  | Amplification ID4 | NA | Forward | 4 | ONT_ID4_fw4 | TGGTCAACGATACAAGCAGAAGACGGCATACGAGAT |
|  |  |  |  | Reverse | 4 | ONT_ID4_rv4 | TGGTCAACGATAAATGATACGGCGACCACCGAGATC |
|  |  | Amplification ID5 | NA | Forward | 4 | ONT_ID5_fw5 | ATCGCACAGTAACAAGCAGAAGACGGCATACGAGAT |
|  |  |  |  | Reverse | 4 | ONT_ID5_rv5 | ATCGCACAGTAAAATGATACGGCGACCACCGAGATC |
|  |  | Amplification ID6 | NA | Forward | 4 | ONT_ID6_fw6 | GTCGTGTAGCCTCAAGCAGAAGACGGCATACGAGAT |
|  |  |  |  | Reverse | 4 | ONT_ID6_rv6 | GTCGTGTAGCCTAATGATACGGCGACCACCGAGATC |
|  |  | Amplification ID7 | NA | Forward | 4 | ONT_ID7_fw7 | AGCGGAGGTTAGCAAGCAGAAGACGGCATACGAGAT |
|  |  |  |  | Reverse | 4 | ONT_ID7_rv7 | AGCGGAGGTTAGAATGATACGGCGACCACCGAGATC |
|  |  | Amplification ID8 | NA | Forward | 4 | ONT_ID8_fw8 | ATCCTTTGGTTCCAAGCAGAAGACGGCATACGAGAT |
|  |  |  |  | Reverse | 4 | ONT_ID8_rv8 | ATCCTTTGGTTCAATGATACGGCGACCACCGAGATC |
| dPCR | Delporte,  et al. Clin Chem. 2025 | RU5 (total HIV) | 518-647 | Forward | NA | RU5_forward | TTAAGCCTCAATAAAGCTTGCC |
|  |  |  |  | Reverse | NA | RU5_reverse | GTTCGGGCGCCACTGCTAGA |
|  |  |  |  | Probe | NA | RU5_probe | CCAGAGTCACACAACAGACGGGCACA |
|  |  | *psi* | 692-797 | Forward | NA | psi_forward | CAGGACTCGGCTTGCTGAAG |
|  |  |  |  | Reverse | NA | psi_reverse | GCACCCATCTCTCTCCTTCTAGC |
|  |  |  |  | Probe | NA | psi_probe | TTTTGGCGTACTCACCAGT |
|  |  | *gag* | 1300-1377 | Forward | NA | gag_forward | ATGTTTTCAGCATTATCAGAAGGA |
|  |  |  |  | Reverse | NA | gag_reverse | TGCTTGATGTCCCCCCACT |
|  |  |  |  | Probe | NA | gag_probe | CCACCCCACAAGATTTAAACACCATGCTAA |
|  |  | *pol* | 2536-2662 | Forward | NA | pol_forward | GCACTTTAAATTTTCCCATTAGTCCTA |
|  |  |  |  | Reverse | NA | pol_reverse | CAAATTTCTACTAATGCTTTTATTTTTTC |
|  |  |  |  | Probe | NA | pol_probe | AAGCCAGGAATGGATGGCC |
|  |  | *env* | 7736-7851 | Forward | NA | env_forward | AGTGGTGCAGAGAGAAAAAAGAGC |
|  |  |  |  | Reverse | NA | env_reverse | GTCTGGCCTGTACCGTCAGC |
|  |  |  |  | Probe | NA | env_probe | CCTTGGGTTCTTGGGA |
|  |  |  |  | Dark probe | NA | env_darkprobe | CC+TTAGGTTCTTAGG+AGC |
| *env* PCR |  | *env* | 6540-7721 | Forward | 1 | ENV1_F1 | GAGGATATAATCAGTTTATGG |
|  |  |  |  | Reverse | 1 | N001_R1 | GGTGGGTGCTAYTCCYADTGG |
|  |  |  | 6542-7721 | Forward | 1 | ENV11_F1 | GGATATAATCAGYYTATGGGA |
|  |  |  |  | Reverse | 1 | N002_R1 | GGTGGGTGCTATTCCTARTGG |
|  |  |  | 6561-7667 | Forward | 2 | ENV33_F2 | GATCAAAGCCTAAARCCATGT |
|  |  |  |  | Reverse | 2 | E7238_R2 | ACTTCTCCAATTGTCCCTCATAT |
|  |  |  | 6561-7663 | Forward | 2 | ENV2_F2 | GATCAAAGCCTAAAGCCATG |
|  |  |  |  | Reverse | 2 | ENV44_R2 | CTCCAATTGTCCYTCATHTYTCC |
|  |  |  | NA | Forward | Sequencing | E6951_F_seq | AGYRCAGTACAATGYACACATGG |
|  |  |  | NA | Forward | Sequencing | E6990_F_seq | TCAACHCAAYTRCTGTTAAATGG |
|  |  |  | NA | Reverse | Sequencing | E7336_R_seq | ATTTCTGGRTCYCCKCCTG |
|  |  |  | NA | Reverse | Sequencing | E7382_R_seq | ATTACARTAGAAAAATTCYCCTCYAC |

**Supplementary Table 5. Proviral counts per participant**
The table provides an overview of the total and unique proviral counts per participant, and the unique counts among the proviral classes.

| **Participant** | **Total** | **Unique** | **Inversion** | **Large deletion** | **Hypermutation** | **Premature stop-codon/ Frameshift** | **PSI/MSD** | **Intact** |
| --- | --- | --- | --- | --- | --- | --- | --- | --- |
| 1 | 10 | 5 | 1 | 2 | 0 | 0 | 2 | 0 |
| 2 | 36 | 30 | 0 | 12 | 9 | 3 | 0 | 6 |
| 3 | 30 | 21 | 1 | 10 | 8 | 1 | 0 | 1 |
| 4 | 4 | 4 | 0 | 2 | 0 | 0 | 1 | 1 |
| 5 | 70 | 65 | 5 | 60 | 0 | 0 | 0 | 0 |
| 6 | 16 | 12 | 0 | 6 | 1 | 1 | 4 | 0 |
| 7 | 27 | 21 | 0 | 11 | 1 | 5 | 0 | 4 |
| 8 | 27 | 27 | 0 | 15 | 6 | 0 | 2 | 4 |
| 9 | 84 | 66 | 1 | 38 | 11 | 1 | 7 | 8 |
| 10 | 46 | 42 | 3 | 25 | 1 | 3 | 4 | 6 |
| 11 | 56 | 55 | 8 | 32 | 5 | 4 | 1 | 5 |
| 12 | 61 | 44 | 0 | 26 | 2 | 9 | 4 | 3 |
| 13 | 62 | 60 | 6 | 36 | 5 | 0 | 9 | 4 |
| 14 | 27 | 25 | 1 | 15 | 2 | 1 | 1 | 5 |
| 15 | 34 | 30 | 0 | 18 | 7 | 0 | 2 | 3 |
| 16 | 17 | 13 | 1 | 8 | 0 | 0 | 1 | 3 |
| 17 | 32 | 29 | 0 | 18 | 1 | 0 | 1 | 9 |
| 18 | 9 | 8 | 1 | 5 | 0 | 0 | 2 | 0 |
| 19 | 33 | 32 | 1 | 20 | 2 | 2 | 3 | 4 |
| 20 | 55 | 47 | 0 | 30 | 8 | 0 | 8 | 1 |
| 21 | 77 | 72 | 0 | 46 | 13 | 5 | 8 | 0 |
| 22 | 28 | 17 | 1 | 11 | 0 | 3 | 2 | 0 |
| 23 | 22 | 17 | 0 | 11 | 5 | 0 | 0 | 1 |
| 24 | 7 | 6 | 0 | 4 | 1 | 0 | 0 | 1 |
| 25 | 18 | 15 | 1 | 10 | 3 | 0 | 1 | 0 |
| 26 | 93 | 79 | 5 | 53 | 9 | 0 | 7 | 5 |
| 27 | 61 | 55 | 3 | 37 | 5 | 0 | 5 | 5 |
| 28 | 46 | 43 | 1 | 29 | 8 | 0 | 4 | 1 |
| 29 | 94 | 90 | 3 | 61 | 3 | 7 | 2 | 14 |
| 30 | 31 | 28 | 0 | 19 | 4 | 2 | 2 | 1 |
| 31 | 102 | 96 | 4 | 66 | 2 | 1 | 15 | 8 |
| 32 | 56 | 49 | 1 | 34 | 9 | 0 | 2 | 3 |
| 33 | 56 | 46 | 1 | 32 | 5 | 1 | 3 | 4 |
| 34 | 17 | 17 | 0 | 12 | 0 | 1 | 3 | 1 |
| 35 | 45 | 41 | 1 | 30 | 4 | 0 | 6 | 0 |
| 36 | 21 | 19 | 0 | 14 | 3 | 0 | 1 | 1 |
| 37 | 44 | 37 | 3 | 28 | 0 | 0 | 1 | 5 |
| 38 | 39 | 34 | 1 | 26 | 2 | 2 | 2 | 1 |
| 39 | 30 | 27 | 1 | 21 | 0 | 1 | 4 | 0 |
| 40 | 22 | 19 | 0 | 15 | 1 | 0 | 2 | 1 |
| 41 | 15 | 15 | 0 | 12 | 2 | 0 | 0 | 1 |
| 42 | 26 | 25 | 1 | 20 | 1 | 0 | 0 | 3 |
| 43 | 52 | 42 | 3 | 34 | 3 | 1 | 1 | 0 |
| 44 | 69 | 58 | 3 | 47 | 3 | 0 | 4 | 1 |
| 45 | 16 | 16 | 0 | 13 | 1 | 2 | 0 | 0 |
| 46 | 55 | 49 | 1 | 40 | 0 | 1 | 5 | 2 |
| 47 | 11 | 11 | 0 | 9 | 0 | 0 | 2 | 0 |
| 48 | 56 | 56 | 5 | 46 | 2 | 0 | 2 | 1 |
| 49 | 36 | 28 | 1 | 23 | 0 | 1 | 1 | 2 |
| 50 | 26 | 23 | 2 | 19 | 0 | 1 | 0 | 1 |
| 51 | 56 | 47 | 0 | 39 | 1 | 0 | 6 | 1 |
| 52 | 52 | 49 | 2 | 41 | 1 | 1 | 4 | 0 |
| 53 | 47 | 43 | 0 | 36 | 1 | 3 | 3 | 0 |
| 54 | 26 | 26 | 2 | 22 | 1 | 0 | 1 | 0 |
| 55 | 52 | 33 | 0 | 28 | 0 | 0 | 4 | 1 |
| 56 | 84 | 80 | 1 | 68 | 1 | 0 | 9 | 1 |
| 57 | 102 | 95 | 1 | 81 | 3 | 1 | 8 | 1 |
| 58 | 26 | 21 | 0 | 18 | 1 | 1 | 1 | 0 |
| 59 | 87 | 77 | 6 | 66 | 0 | 1 | 4 | 0 |
| 60 | 214 | 201 | 11 | 175 | 0 | 7 | 4 | 4 |
| 61 | 24 | 24 | 0 | 21 | 1 | 0 | 0 | 2 |
| 62 | 270 | 263 | 11 | 233 | 0 | 8 | 5 | 6 |
| 63 | 40 | 37 | 1 | 33 | 0 | 0 | 2 | 1 |
| 64 | 46 | 39 | 2 | 35 | 0 | 0 | 2 | 0 |
| 65 | 120 | 110 | 4 | 99 | 2 | 3 | 1 | 1 |
| 66 | 150 | 120 | 3 | 110 | 1 | 0 | 5 | 1 |
| 67 | 69 | 61 | 1 | 56 | 2 | 0 | 2 | 0 |
| 68 | 34 | 25 | 0 | 23 | 0 | 0 | 2 | 0 |
| 69 | 67 | 64 | 3 | 59 | 0 | 0 | 2 | 0 |
| 70 | 17 | 14 | 0 | 13 | 0 | 0 | 0 | 1 |
| 71 | 34 | 34 | 2 | 32 | 0 | 0 | 0 | 0 |
| 72 | 51 | 45 | 2 | 43 | 0 | 0 | 0 | 0 |
| 73 | 155 | 135 | 6 | 129 | 0 | 0 | 0 | 0 |
| 74 | 25 | 25 | 1 | 24 | 0 | 0 | 0 | 0 |
| 75 | 27 | 26 | 0 | 25 | 0 | 0 | 1 | 0 |
| 76 | 75 | 67 | 1 | 65 | 0 | 0 | 1 | 0 |
| 77 | 38 | 38 | 1 | 37 | 0 | 0 | 0 | 0 |
| 78 | 86 | 80 | 2 | 78 | 0 | 0 | 0 | 0 |
| 79 | 91 | 84 | 2 | 82 | 0 | 0 | 0 | 0 |
| 80 | 133 | 121 | 2 | 119 | 0 | 0 | 0 | 0 |
| 81 | 195 | 183 | 1 | 182 | 0 | 0 | 0 | 0 |
| 82 | 3 | 3 | 0 | 3 | 0 | 0 | 0 | 0 |
| 83 | 4 | 4 | 0 | 4 | 0 | 0 | 0 | 0 |

**Supplementary Table 6. Comprehensive overview of HIV drug resistance mutations detected per participant and individual proviruses**The table lists all mutations detected by HIV-PULSE for each participant, allowing identification of combinations of mutations across different drug classes that occur across proviruses and on the same proviral sequence.

*See SupplementaryTable6.xlsx*

**Supplementary Table 7. In silico bNAb sensitivity predictions among proviral classes**bNAb sensitivity was predicted by bNAb-ReP tool of 2,612 unique proviral *env* sequences. For each proviral class, the proportion of sequences that were predicted to get neutralized or be resistant is displayed in the table.

|  | **Intact** | | **Large deletions** | | **PSI/MSD defects** | | **Premature stop-codon/ Frameshift** | |
| --- | --- | --- | --- | --- | --- | --- | --- | --- |
|  | Neutralizing | Resistant | Neutralizing | Resistant | Neutralizing | Resistant | Neutralizing | Resistant |
| All bNAbs | 83·2 (55·5%) | 66·8 (44·5%) | 568·5 (24·5%) | 1756·5 (75·5%) | 113·8 (55·5%) | 91·2 (44·5%) | 43·3 (52·8%) | 38·7 (47·2%) |
| CD4bs | 104·3 (69·5%) | 45·7 (30·5%) | 711·7 (30·6%) | 1613·3 (69·4%) | 145·3 (70·9%) | 59·7 (29·1%) | 51·8 (63·2%) | 30·2 (36·8%) |
| gp120-g41 interface | 61·0 (40·7%) | 89·0 (59·3%) | 424·8 (18·3%) | 1900·3 (81·7%) | 84·8 (41·3%) | 120·3 (58·7%) | 32·0 (39·0%) | 50·0 (61·0%) |
| MPER | 98·5 (65·7%) | 51·5 (34·3%) | 671·5 (28·9%) | 1653·5 (71·1%) | 129·0 (62·9%) | 76·0 (37·1%) | 41·5 (50·6%) | 40·5 (49·4%) |
| V1/V2 loop | 33·6 (22·4%) | 116·4 (77·6%) | 321·3 (13·8%) | 2003·7 (86·2%) | 52·7 (25·7%) | 152·3 (74·3%) | 24·6 (30·0%) | 57·4 (70·0%) |
| V3 glycan | 10·.7 (68·5%) | 47·3 (31·5%) | 635·4 (27·3%) | 1689·6 (72·7%) | 133·5 (65·1%) | 71·5 (34·9%) | 52·8 (64·4%) | 29·2 (35·6%) |

**Supplementary Table 8. Assessment of the Gag^77-85^ epitope variants**Across 1,636 unique *gag* sequences, the prevalence of Gag^77-85^ epitope variants was assessed. For each target epitope, the prevalence in intact sequences is displayed in the last column. EC_50_, half-maximal effective concentration.

| **Target sequence** | **Relative EC_50_** | **Prevalence all sequences** | **Prevalence intact sequences** |
| --- | --- | --- | --- |
| SLYNTVATL | 1 | 16·2% | 19·3% |
| SLYNT**I**A**V**L | 0·77 | 4·6% | 10·0% |
| SLYNT**I**ATL | 0·60 | 13·8% | 12·0% |
| SL**F**NT**I**A**V**L | 2·92 | 3·8% | 6·0% |
| SLYNTVA**V**L | 1·68 | 9·2% | 17·3% |
| SL**F**NT**I**ATL | 1·19 | 4·5% | 2·0% |
| SL**F**NTVATL | 2·28 | 16·3% | 12·0% |
| SL**F**NTVA**V**L | 6·28 | 3·4% | 2·0% |
| Others | NA | 27·9% | 19·3% |

**Supplementary Table 9. Assessment of the Gag^77-85^ epitope variants with unknown IMC-M113V binding efficiency**

| **Target sequence** | **Relative EC_50_** | **Prevalence all sequences** | **Prevalence intact sequences** |
| --- | --- | --- | --- |
| S**V**YNTVATL | NA | 21·1% | 0·0% |
| SL**H**NTVATL | NA | 16·9% | 13·8% |
| SL**F**N**AI**A**V**L | NA | 14·9% | 3·4% |
| SLYNTVAT**I** | NA | 10·1% | 0·0% |
| SL**F**N**A**VA**V**L | NA | 9·9% | 3·4% |
| SLYN**A**VA**V**L | NA | 4·8% | 31·0% |
| SLYN**LI**ATL | NA | 2·6% | 0·0% |
| S**VF**NT**I**A**V**L | NA | 2·2% | 6·9% |
| SL**W**NTVATL | NA | 2·0% | 3·4% |
| SLYN**L**VA**V**L | NA | 2·0% | 6·9% |
| S**VF**N**AI**A**V**L | NA | 1·8% | 10·3% |
| S**VF**NTVA**V**L | NA | 1·8% | 10·3% |
| SLYN**L**VATL | NA | 1·5% | 0·0% |
| SLYN**AI**A**V**L | NA | 1·3% | 0·0% |
| S**I**YN**L**VA**V**L | NA | 1·1% | 3·4% |
| SLYN**A**VATL | NA | 0·9% | 0·0% |
| SL**F**N**A**V**TV**L | NA | 0·7% | 0·0% |
| SL**F**NTVAT**I** | NA | 0·7% | 3·4% |
| SL**H**NTVA**V**L | NA | 0·4% | 0·0% |
| SLYNT**T**A**V**L | NA | 0·4% | 0·0% |
| **A**L**F**NTVATL | NA | 0·2% | 0·0% |
| **C**LYNTVA**V**L | NA | 0·2% | 0·0% |
| SL**F**N**LI**ATL | NA | 0·2% | 0·0% |
| SL**F**NT**I**A**V** | NA | 0·2% | 0·0% |
| SL**F**NT**IVV**L | NA | 0·2% | 0·0% |
| SL**F**NTVA**A**L | NA | 0·2% | 3·4% |
| SL**H**N**A**VA**V**L | NA | 0·2% | 0·0% |
| SL**H**NT**I**A**V**L | NA | 0·2% | 0·0% |
| SLYNT**I**AT**P** | NA | 0·2% | 0·0% |
| SLYNT**I**A**V** | NA | 0·2% | 0·0% |
| SLYNT**IS**TL | NA | 0·2% | 0·0% |
| SLYNT**ITV**L | NA | 0·2% | 0·0% |
| SLYNTV**VV**L | NA | 0·2% | 0·0% |
| S**SF**NT**I**ATL | NA | 0·2% | 0·0% |

The prevalence of Gag^77-85^ epitope variants with unknown IMC-M113V binding efficiency was assessed among all and intact sequences with unknown EC50. The amino acid switch is highlighted in bold compared to the cognate sequence SLYNTVATL. EC_50_, half-maximal effective concentration.
